# ZnT3 expression levels are down-regulated in the brain of *Mcoln1* knockout mice

**DOI:** 10.1186/s13041-019-0446-3

**Published:** 2019-03-26

**Authors:** Jonathan Chacon, Lauren Rosas, Math P. Cuajungco

**Affiliations:** 1 Department of Biological Science, California State University Fullerton, 800 N. State College Blvd., Fullerton, CA 92831 USA; 2 Center for Applied Biotechnology Studies, California State University Fullerton, Fullerton, CA 92831 USA

**Keywords:** Zinc, ZnT3, Slc30a3, AP3, Ap3d1, TRPML1, Mucolipidosis IV, Neurodegenerative disorders

## Abstract

**Aim:**

Zinc is a critical divalent cation in mammalian brain, but its concentration must be strictly-controlled. Within certain subsets of glutamatergic neurons, ZnT3 (encoded by the *Slc30a3* gene) facilitates the transport and storage of zinc in synaptic vesicles. It has been previously reported that *Slc30a3* mRNA levels are perturbed in numerous neurodegenerative disorders. Given the growing evidence of zinc dysregulation in another neurodegenerative disease known as Mucolipidosis IV (MLIV), we hypothesized that abnormal ZnT3 expression would be observed in the brain of MLIV mouse model. Elucidating the link between abnormal ZnT3 and zinc levels could reveal the neuropathological correlates between MLIV and other age-related brain disorders.

**Methods:**

Total RNAs from cortical tissues of Mucolipin-1 knockout (*Mcoln1*^*−/−*^ KO) and *Mcoln1*^*+/+*^ wild-type (WT) littermate control mice were analyzed for differential gene expression (DGE) using RNA sequencing (RNA-seq). Real-time quantitative PCR (qPCR) and Western blot techniques were used to validate the data.

**Results:**

RNA-seq analysis showed a marked decrease in baseline levels of *Slc30a3* mRNA in *Mcoln1*^*−/−*^ mice. Real-time qPCR and Western blot analyses confirmed that *Slc30a3* transcripts and its protein levels were significantly reduced. Our observations add MLIV to a growing list of neurodegenerative diseases that parallels abnormal ZnT3 expression with zinc dyshomeostasis.

**Electronic supplementary material:**

The online version of this article (10.1186/s13041-019-0446-3) contains supplementary material, which is available to authorized users.

Zinc is a critical trace element for life; however, its involvement in various neurodegenerative diseases is well document [1]. Within a subset of glutamatergic neurons, facilitated zinc transport into synaptic vesicles is entirely achieved through ZnT3 (encoded by the *Slc30a3* gene) [2]. Interestingly, aberrant ZnT3 expression levels in the brain appear to be a common feature in Alzheimer’s disease, Lewy-body dementia, and amyotrophic lateral sclerosis, in which abnormal cerebral zinc levels have been implicated in disease process [3–5]. Recent evidence from our laboratory suggests that zinc dyshomeostasis plays a role in the pathogenesis of Mucolipidosis IV (MLIV) [6, 7], which has since been corroborated by another group [8]. MLIV is caused by the loss of TRPML1 function, which is a lysosomal cation channel encoded by the *MCOLN1* gene. The existing *Mcoln1*^*−/−*^ KO mice have been shown to mimic MLIV disease phenotype [9], and is thus an excellent model to dissect pathological processes connected with zinc dyshomeostasis.

A detailed description of experimental methods, including the RNA-seq approach (Additional file 1: Table S1) and real-time qPCR primers (Additional file 1: Table S2) can be found in Additional file 1. Baseline transcript expression levels from *Mcoln1*^*−/−*^ KO and *Mcoln1*^*+/+*^ WT littermate control mice (aged 2–3 months) were adjusted for gene length and library size using the Transcripts Per Million (TPM) normalization. We generated a list of DGE using Galaxy’s DeSeq2 analysis to further corroborate the TPM data (Additional file 2: Table S3). Gene ontology (GO) analysis of the RNA-seq data revealed significant DGE under the “zinc ion transmembrane transporter activity” category. This led us to look into *Slc30a3* mRNAs levels. Despite the variations within and between KO and WT samples, marked decrease in *Slc30a3* transcripts among the *Mcoln1*^*−/−*^ KO brain samples was consistently observed (Fig. 1a). DeSeq2 analysis showed a two-fold down-regulation of *Slc30a3* expression in KO brain samples (Additional file 2: Table S3, log2[FC] = − 0.8 ± 0.3, *p-value* = 0.02). The list also befittingly showed the reduction of *Mcoln1* transcripts (log2[FC] = − 0.7 ± 0.3, *p-value* = 0.03) as a consequence of exons 3 and 4 exclusion caused by the transgene. Indeed, the sequencing coverage for *Mcoln1* gene is in agreement with the excised exons that produced the KO phenotype [9] (Additional file 1: Figure S1). Validation with real-time qPCR confirmed the reduced *Slc30a3* transcripts (Fig. 1b). Integrated density value (IDV) analysis (Fig. 1c) of Western blot experiments (Fig. 1d) further confirmed the RNA-seq and qPCR data. Although a sex-specific negative regulation of ZnT3 and Ap3d1 (a subunit of the AP3 complex) expression levels have been reported in mice exposed to incremental doses of the estrogen analog estradiol [10], our data did not show such an effect since the animals were fed a standard chow. Nevertheless, this report prompted us to analyze the *Ap3d1* transcripts despite not identifying *Ap3d1* on the DGE list. Using real-time qPCR, we found a reduction in *Ap3d1* mRNA levels (Additional file 1: Figure S2), which parallels the reduction of *Slc30a3* mRNA and protein levels observed in the current study (Fig. 1).

**Fig. 1:**
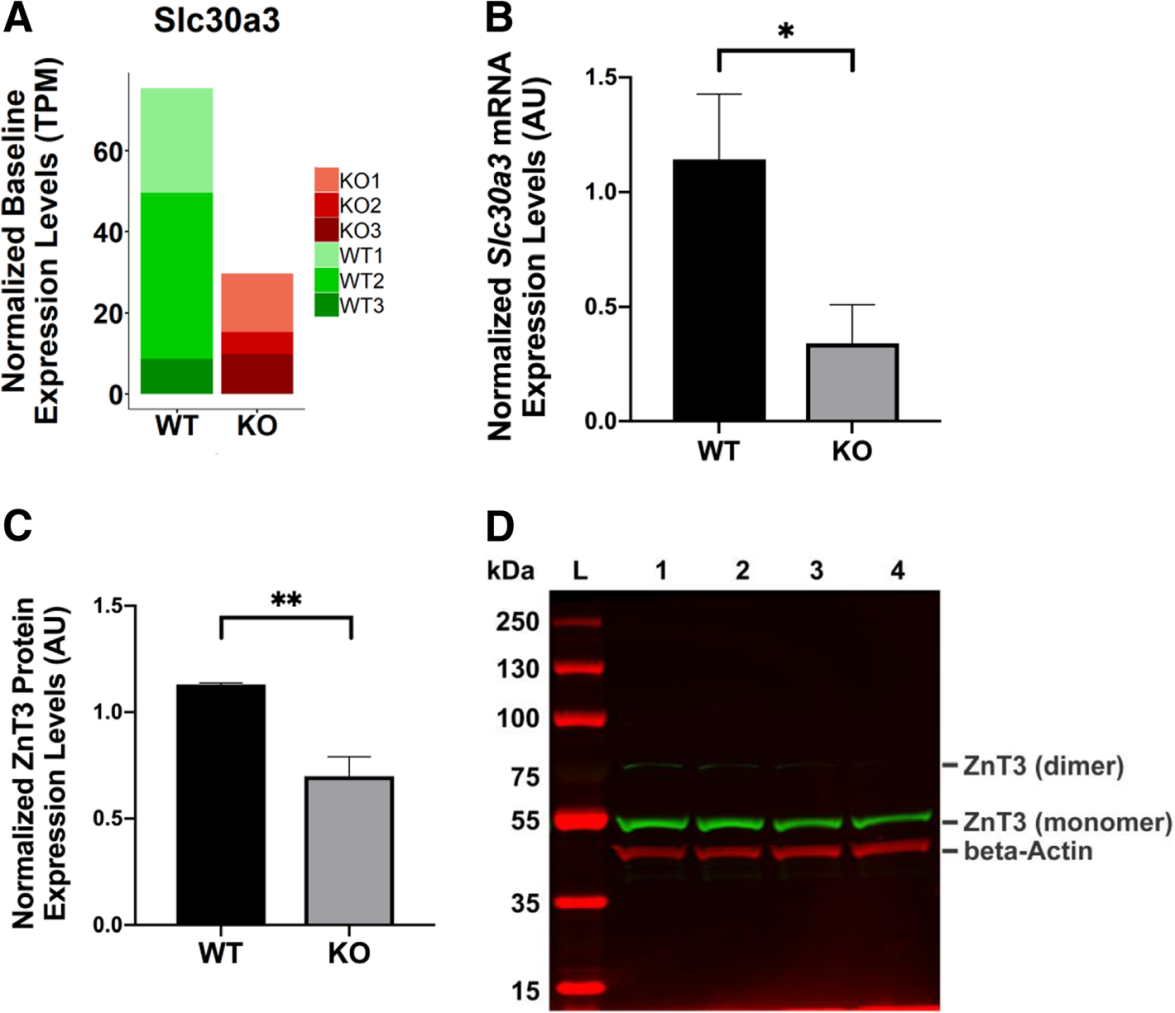
Baseline *Slc30a3* mRNA and Slc30a3 (ZnT3) protein expression levels. **a** Transcriptomic analysis of brain tissues from *Mcoln1*^*−/−*^ KO mice (KO1-KO3, *n* = 3) and *Mcoln1*^*+/+*^ WT (WT1-WT3, n = 3) littermate control mice. **b** Real-time qPCR analysis of relative *Slc30a3* mRNA expression levels from brain tissues taken from *Mcoln1*^*−/−*^ KO and *Mcoln1*^*+/+*^ WT littermate control mice. The qPCR experiments were done in triplicate wells, normalized using *18S rRNA*, and analyzed using the Standard Curve method. The data are represented as mean ± SD (*p < 0.05, Student’s *t*-test, paired, *n* ≥ 3). **c** Integrated density value analysis of Slc30a3 (ZnT3) protein bands normalized with beta-Actin bands from two independent Western blot experiments. The relative Slc30a3 protein expression levels in *Mcoln1*^*−/−*^ KO-A and KO-B mouse brains are significantly reduced in comparison to *Mcoln1*^*+/+*^ WT-A mouse brain (**p < 0.01, Student’s *t*-test, paired, *n* ≥ 2). **d** Representative Western blot image of ZnT3 (*green*; monomer: ~ 42 kDa; dimer: ~ 84 kDa) and beta-Actin proteins (*red*; ~ 41 kDa). Each lane corresponds to: L, protein ladder; 1, *Mcoln1*^*+/+*^ WT-A control brain; 2, *Mcoln1*^*+/−*^ heterozygote control brain; 3, *Mcoln1*^*−/−*^ KO-A brain; and 4, *Mcoln1*^*−/−*^ KO-B brain. The blot was probed with anti-beta-Actin mouse monoclonal antibody and anti-ZnT3 rabbit polyclonal antibody, and imaged using LICOR Odyssey Sa infrared scanner at 700 nm and 800 nm channels, respectively

MLIV disease phenotypes such as abnormal gait, hind-limb paralysis, and mortality are typically observed between six and 9 months [9]. The significant reduction of *Slc30a3*/ZnT3 expression in *Mcoln1*^*−/−*^ KO brain tissue suggests that it may be used as a biomarker. Our previous reports that zinc dyshomeostasis may be a key pathological event that initiates neuronal death and contributes to progressive neurodegeneration in MLIV have been gaining ground and are independently confirmed by others. Whether the distinct reduction of *Slc30a3*/ZnT3 expression in *Mcoln1*^*−/−*^ KO mice is a cause or consequence of the disease remains to be elucidated. However, the concomitant downregulation of *Ap3d1*, a key AP3 complex subunit involved in co-targeting ZnT3 protein and vesicular glutamate transporter 1 (Vglut1) to synaptic vesicles [11], suggests that the former argument may not be the case. Noteworthy is that oxidative stress has been implicated in MLIV [12] and other neurodegenerative disorders [1], and that reactive oxygen [13] and nitrogen [14] species have been shown to uncontrollably release chelatable zinc. Therefore, it may be that the reductions in *Slc30a3*/ZnT3 expression could reflect a cytoprotective role to limit the neurotoxic release of glutamatergic zinc-rich vesicles. The recent observations of augmented glutamate exocytosis in *Mcoln1*^*−/−*^ KO neurons [15] and enhanced loading of glutamate into zinc-rich vesicles by ZnT3 and Vglut1 proteins [11] lend further credence to the possibility that decreased S*lc30a3*/ZnT3 expression in certain brain disorders may be a negative feedback regulation to prevent or minimize both glutamate and zinc-induced cytotoxicity. Future proteomic studies using *Mcoln1*^*−/−*^ brain tissues are warranted to yield better insight into the mechanistic processes that underlie zinc dyshomeostasis in MLIV and other neurodegenerative diseases.

## Additional files

Additional file 1: Materials and methods. **Table S1.** Basic demographics, sample IDs, and RNA-seq reads for each mouse sample. **Table S2.** Tabulated list of real-time qPCR primers used to validate specific RNA-seq data. **Figure S1**. RNA-seq coverage for exons within the major mouse *Mcoln1* isoform. **Figure S2**. Baseline *Ap3d1* transcript levels of individual brain tissues taken from *Mcoln1*^*–/–*^ knockout (KO1-KO3, *n* = 3) and *Mcoln1*^*+/+*^ wild-type (WT1-WT3, n = 3) littermate control mice. (DOCX 5087 kb)

Additional file 2: **Table S3.** Tabulated list of differentially expressed genes between *Mcoln1*^*−/−*^ KO and *Mcoln1*^*+/+*^ WT control samples using DESeq2. (DOCX 150 kb)

## Abbreviations

AP3: adaptor protein 3
DGE: differential gene expression
Mcoln1: mucolipin-1
qPCR: quantitative polymerase chain reaction
RNA-seq: RNA sequencing
Slc30a3: solute carrier 30 type a3
TPM: transcripts per million
Vglut1: vesicular glutamate transporter 1
ZnT3: zinc transporter 3
