# Supplementary material for: ZnT3 expression levels are down-regulated in the brain of Mcoln1 knockout mice
Source: Mol Brain. 2019 Mar 26;12:24. doi: 10.1186/s13041-019-0446-3 (PMC6434829; doi:10.1186/s13041-019-0446-3)
Supplement: Supplementary file 1 — Materials and methods. Table S1. Basic demographics, sample IDs, and RNA-seq reads for each mouse sample. Table S2. Tabulated list of real-time qPCR primers used to validate specific RNA-seq data. Figure S1. RNA-seq coverage for exons within the major mouse Mcoln1 isoform. Figure S2. Baseline Ap3d1 transcript levels of individual brain tissues taken from Mcoln1–/– knockout (KO1-KO3, n = 3) and Mcoln1+/+ wild-type (WT1-WT3, n = 3) littermate control mice. (DOCX 5087 kb) [file 13041_2019_446_MOESM1_ESM.docx]

**Additional file 1**

**ZnT3 expression levels are down-regulated in the brain of *Mcoln1* knockout mice**

**Jonathan Chacon, Lauren Rosas, and** **Math P. Cuajungco**

Department of Biological Science, and Center for Applied Biotechnology Studies, California State University Fullerton, Fullerton, CA, 92831, USA

**Corresponding author:**

Math P. Cuajungco, PhD

California State University Fullerton

Dept. of Biological Science

800 N. State College Blvd.

Fullerton, CA 92831

Tel: 657-278-8522

Fax: 657-278-3426

Jonathan Chacon, e-mail: jchacon27@csu.fullerton.edu

Lauren Rosas, e-mail: lrosas@csu.fullerton.edu

Math P. Cuajungco, e-mail: mcuajungco@fullerton.edu

**This file includes:**

Materials and Methods

Table S1

Table S2

Figure S1

Figure S2

**MATERIALS AND METHODS**

*Tissue Samples*

Tissues from the cerebral cortex were obtained from *Mcoln1^–/–^* knockout (KO; n = 3, labelled KO1-KO3) and *Mcoln1^+/+^* wild-type (WT) littermate control mice (n = 3, labelled WT1-WT3). Table S1 shows the demographic information of the samples. The brain tissues were dissected into pieces, and were flash frozen in liquid nitrogen for storage at –80°C. A portion of these frozen brain tissues were used in our previous study showing zinc dyshomeostasis in *Mcoln1^–/–^* KO mouse brain [[1](#_ENREF_1)], while the remaining samples were stored for RNA extraction that were subsequently used in the current study.

To get more tissue samples for subsequent validation experiments, additional brain tissues were obtained from one new *Mcoln1^+/+^* WT littermate control (designated as WT-A; female, 3 months old) and two new *Mcoln1^–/–^* KO (designated as KO-A and KO-B; both males, 3 months old). For these new samples, we collected one cortical hemisphere for use in real-time qPCR experiments and the other hemisphere was used for Western blot analysis to validate the transcriptomic data. For Western blot experiments, we also used brain tissue from *Mcoln1^+/–^* heterozygote to serve as another control sample.

*RNA Extraction, Analysis, and cDNA Library Synthesis*

To extract and purify total RNA, the frozen brain tissues were first pulverized using sterile mortar and pestle kept on dry ice. The tissue powder was collected into a microcentrifuge tube, and homogenized using TRI-zol^®^ RNA isolation reagent (Life Technologies, Carlsbad CA). The total RNA samples were then DNAse-treated, purified, eluted in sterile water, quantified using Nanodrop 1000 (ThermoFisher Scientific, Waltham MA), and then stored at –80°C until further use.

The RNA sample quality was determined using Agilent 2100 Bioanalyzer, which provided specific RNA integrity number (RIN) for each sample. The cDNA library was created using the Ovation^®^ Human FFPE RNA-seq Multiplex System kit (NuGen Technologies, San Carlos CA), following the manufacturer’s recommendations for low input RNA. To deplete ribosomal RNA (rRNA), the Insert Dependent Adaptor Cleavage (InDA-C) method was used, which utilized magnetic bead purification and selective InDA-C primers specific for mouse rRNA, which was generously provided by NuGen Technologies. After PCR amplification, the rRNA-depleted cDNA library was sequenced with single-read using Illumina^®^ HiSeq 2500 by the Genomics High Throughput Facility at the University of California, Irvine.

*Transcriptome Assembly and Differential Gene Expression (DGE) Analysis*

*Mcoln1^–/–^* KO and *Mcoln1^+/+^* WT mouse brain transcriptomes were assembled and aligned using the *Mus musculus* reference genome (GRCm38) with Lasergene^®^ SeqMan NGen software v.15.0 (DNAStar, Madison WI). Due to inter-subject variability possibly contributed by differences in RNA Integrity Number (RIN) within and between *Mcoln1^–/–^* KO and WT RNA samples, the transcript profiles were analyzed individually. Analyses of individual samples within the *Mcoln1^–/–^* KO and WT group, as well as between grouping, have allowed us to identify specific trends or potential outliers in the data. Sample demographics, identification (ID), RIN, and total sequence reads are shown in Table S1. Intra-group sample IDs were arranged according to descending RIN or total sequence reads. Reads were assembled using features as transcripts, and pseudogenes were excluded from all transcriptome assemblies. Transcript expression was quantified using the Reads per Kilobase per Million (RPKM), a default normalization method of Lasergene’s SeqMan NGen v.15.0. The total transcripts were adjusted for inter-subject proportionality differences using the Transcripts Per Million (TPM) metric. Sequences were defined using BestRefSeq and Gnomon gene prediction tools (for information on NCBI annotation pipeline, see link: [www.ncbi.nlm.nih.gov/genome/annotation_euk/process/](http://www.ncbi.nlm.nih.gov/genome/annotation_euk/process/)).

To determine DGE and further validate Lasergene outputs, *Mcoln1^+/+^* WT and *Mcoln1^–/–^* KO sample reads were aligned to the *M. musculus* (mm10) reference genome using Galaxy’s (https://usegalaxy.org) HISAT2 (v2.27) alignment program. Gene expression was measured within alignment files using FeatureCounts (v1.6.2) and DGE was computed using Galaxy’s web-based DESeq2 (v2.11.40) and edgeR (v3.20.7) tools with multiple-hypothesis testing being achieved using Benjamani-Hochberg and false-discovery rate (FDR) correction, respectively. Gene sets were then filtered for significance using the p-value and Gene IDs were run through the Database for Annotation, Visualization, and Integrated Discovery (DAVID, v6.8) to obtain official gene symbols (https://david.ncifcrf.gov).

*Real-time Quantitative Polymerase Chain Reaction (qPCR) Validation*

We followed the Minimum Information for Publication of Real-time qPCR Experiments (MIQE) guidelines [[2](#_ENREF_2)] and used the Standard Curve method to analyze the relative transcript expression of down-regulated genes related to zinc handling and transport. Standard curves for each qPCR reaction were established using pooled cDNA taken from each brain RNA sample. All qPCR experiments were run in triplicates using the SensiMix SYBR No-ROX master mix (Bioline USA, Taunton, MA). Mouse *18S* rRNA was used as a gene reference for normalization of relative starting quantities of each sample. We used a CFX96 thermocycler (Bio-Rad, Hercules, CA) with the following standard parameters: 10 minutes at 95°C for initial denaturation; 40 cycles for 15 seconds at 95°C for denaturation, 30 seconds at 62°C for annealing, and 30 seconds at 72°C for extension. Table S2 outlines relevant MIQE parameters regarding the primer sets used in this study.

For qPCR validation of *Slc30a3* transcripts, sample KO2 was excluded from the qPCR analysis following unreliable results from two different primer sets tested. Thus, we used a total of three biological replicates from *Mcoln1^–/–^* KO and four biological replicates *Mcoln1^+/+^* WT for the qPCR analysis. For qPCR validation of *Ap3d1* transcripts, cDNA samples for KO3 and WT1 were not sufficient for use in the experiments and so we used a total of four biological replicates from *Mcoln1^–/–^* KO and three biological replicates for *Mcoln1^+/+^* WT for the qPCR analysis.

We used Prism version 8 (Graphpad, San Diego, CA) to calculate statistical significance between relative mRNA expression levels (Student’s *t*-test, paired, significance level set at p-value < 0.05) and to generate a graphical image of the data.

*Western Blot Analysis*

For Western blot experiments, we used brain tissues from *Mcoln1^–/–^* KO-A and KO-B mice, as well as control samples from *Mcoln1^+/+^* WT-A and *Mcoln1^+/–^* heterozygote mice. We included a heterozygote mouse brain sample as one of the controls in the experiments because this genotype does not exhibit disease phenotype, and to show that the observed reduction of ZnT3 expression is solely due to the KO or disease phenotype. The frozen brain tissues were pulverized, collected into a microcentrifuge tube, and homogenized using a standard lysis buffer (50 mM Tris-HCl, 150 mM NaCl, 1% Nonidet P-40, 1 mM EDTA, 0.25% sodium deoxycolate, 0.1% sodium dodecyl sulfate [SDS]) with added 1X Proteoblock protease inhibitor cocktail (Sigma-Aldrich, St. Louis, MO) and freshly made 1 mM phenylmethanesulfonyl fluoride (PMSF). The homogenates were sonicated (20 kHz) for 1 minute (10 second pulses with 5 second wait) and the samples were placed inside a 4°C room rotating for one hour. The homogenates were centrifuged at 12,000 rpm inside a 4°C room for 30 minutes. The supernatant was collected for total protein quantification using bicinchoninic acid (BCA) assay.

Total protein samples (50 μg) were resolved on 4-12% Bis-Tris NuPAGE gel (ThermoFisher), transferred onto a nitrocellulose membrane, and processed for Western blot. We purchased an anti-Slc30a3 polyclonal antibody (pAb) from Sigma-Aldrich (SAB2105534) and another one from ThermoFisher (PA5-77769). For loading and normalization control, we purchased anti-beta-Actin monoclonal antibody (mAb) from Sigma-Aldrich (A1978). The blots were incubated with Odyssey blocking buffer (LI-COR, Lincoln, NE) in Tris-buffered saline (TBS) for one hour at room temperature. After the blots were washed 3x with TBS plus Tween-20 (TBST), one set of blots were simultaneously incubated with Sigma-Aldrich’s anti-Slc30a3 pAb (1:500) and anti-beta-Actin pAb (1:5000), since both antibodies are from different species. Another set of blots were co-incubated with ThermoFisher’s anti-Slc30a3 pAb (1:500) and Sigma’s anti-beta-Actin mAb (1:5000). The blots were then washed 3x with TBST and incubated with LI-COR’s anti-mouse infrared (IR)-Dye 680LT (1:15000) and anti-rabbit IR-Dye 800CW (1:15000). The blots were washed 3x with TBST and then scanned with LI-COR’s Odyssey Sa IR scanner using both 700 and 800 channels. The images were saved for integrated density value (IDV) analysis.

We used LI-COR’s Image Studio Lite to obtain the IDVs of each Slc30a3 and beta-Actin protein bands to calculate relative protein expression levels. We used the software’s background subtraction tool on all images prior to IDV analysis. To automatically generate a rectangle and calculate mean IDV for each band, the software required the user to click on the protein band. A tabulated fluorescence signal for each channel (red, green, and blue) was produced and the mean IDV was obtained. The mean IDV was then normalized by taking the ratio between Slc30a3 and beta-Actin values. We used Prism version 8 to determine statistical significance of the IDV (Student’s *t*-test, paired, significance level set at p-value < 0.05) and to create a graphical image of the data.

**Table S1.** Basic demographics, sample IDs, and RNA-seq reads for each mouse sample. Intra-group sample IDs are assigned in order of descending RIN values and total sequence reads.

|  | **Wild-type** | | | |  |  | ***Mcoln1^–/–^*** | | | |
| --- | --- | --- | --- | --- | --- | --- | --- | --- | --- | --- |
| Sample ID | | Age / Sex | RIN | Total Reads |  | Sample ID | | Age / Sex | RIN | Total Reads |
| WT1 | | 2 mo. / F | 9 | 30,333,757 |  | KO1 | | 3 mo. / F | 8 | 35,651,630 |
| WT2 | | 3 mo. / F | 9 | 27,637,423 |  | KO2 | | 2 mo. / F | 6 | 32,445,278 |
| WT3 | | 3 mo. / M | 8 | 25,335,469 |  | KO3 | | 3 mo. / F | 5 | 29,512,084 |
|  | |  |  |  |  |  | |  |  |  |
| Mean Reads | | |  | 27,768,883 |  | Mean Reads | | |  | 32,536,331 |

**Table S2.** Tabulated list of real-time qPCR primers used to validate specific RNA-seq data. The primers were designed using the online qPCR assay design tools from Integrated DNA Technologies (Coralville, IA) website and commercially synthesized by the company.

| **Gene Name** | **Primer Sequence (5’ → 3’)** | **Tm** | **Efficiency** | **R^2^** |
| --- | --- | --- | --- | --- |
| *Slc30a3* | Fwd: CGATCCTCATCTACTTCAAGCC  Rev: AAATTCCACACTCCGAGGG | 62 °C | 100 | 1.00 |
| *Ap3d1* | Fwd: AAGATGGTCAAGGGCAGTATC  Rev: ATGCACTGGGAGATGTACTTG | 62 °C | 100 | 0.99 |
| *18S* rRNA | Fwd: CGGCACTTTCGATGGTAGTCGCCG  Rev: GGATGTGGTAGCCGTTTCTCAG | 62 °C | 95 | 0.99 |

**
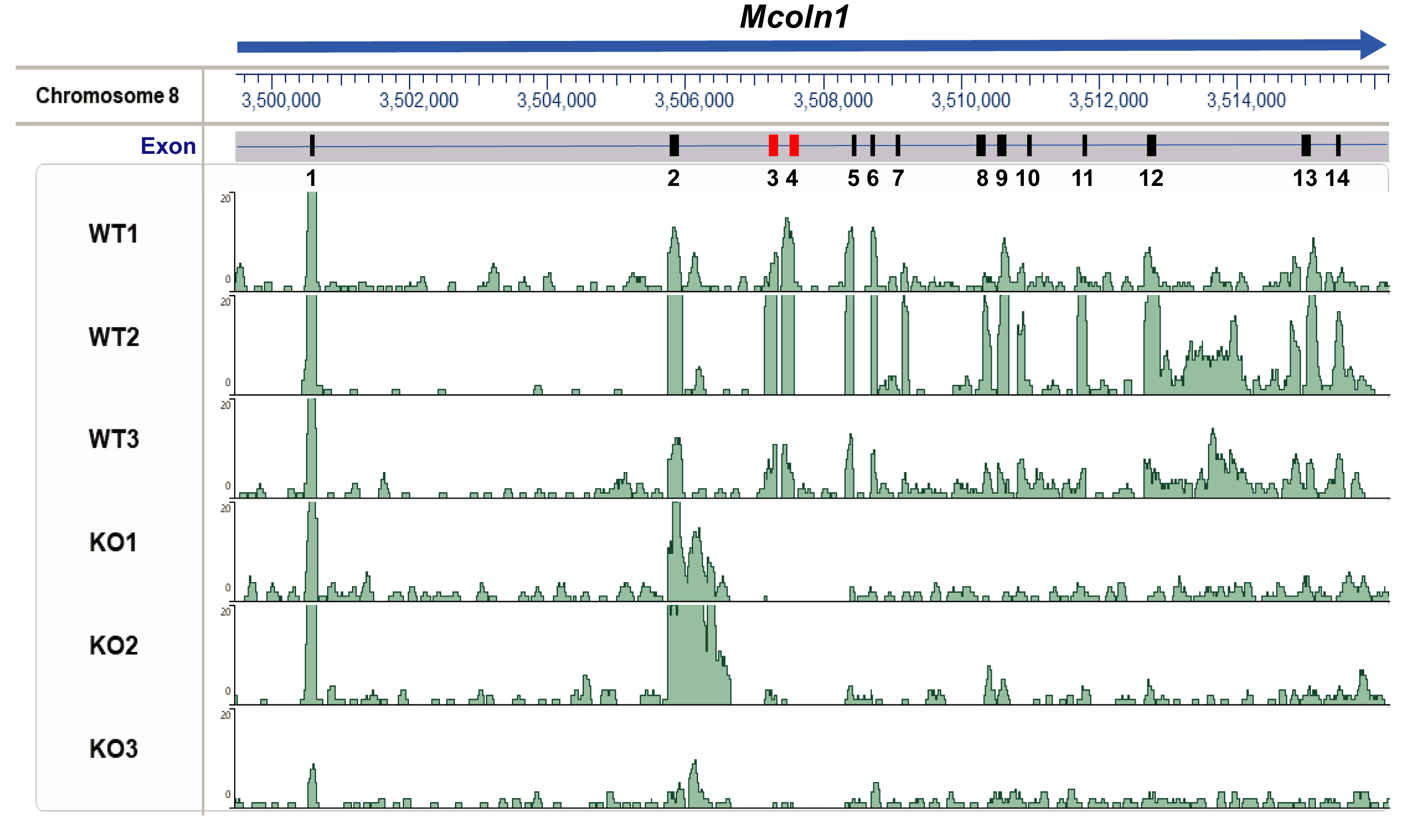
Figure S1**. RNA-seq coverage for exons within the major mouse *Mcoln1* isoform. Exon coverage was visualized using Lasergene’s GenVisionPro software v.15.0. The marked reductions observed in *Mcoln1^–/–^* KO read coverage of exons 3-4 (red bar) represent the region of exon excision as previously reported [[3](#_ENREF_3)].

| **A**  **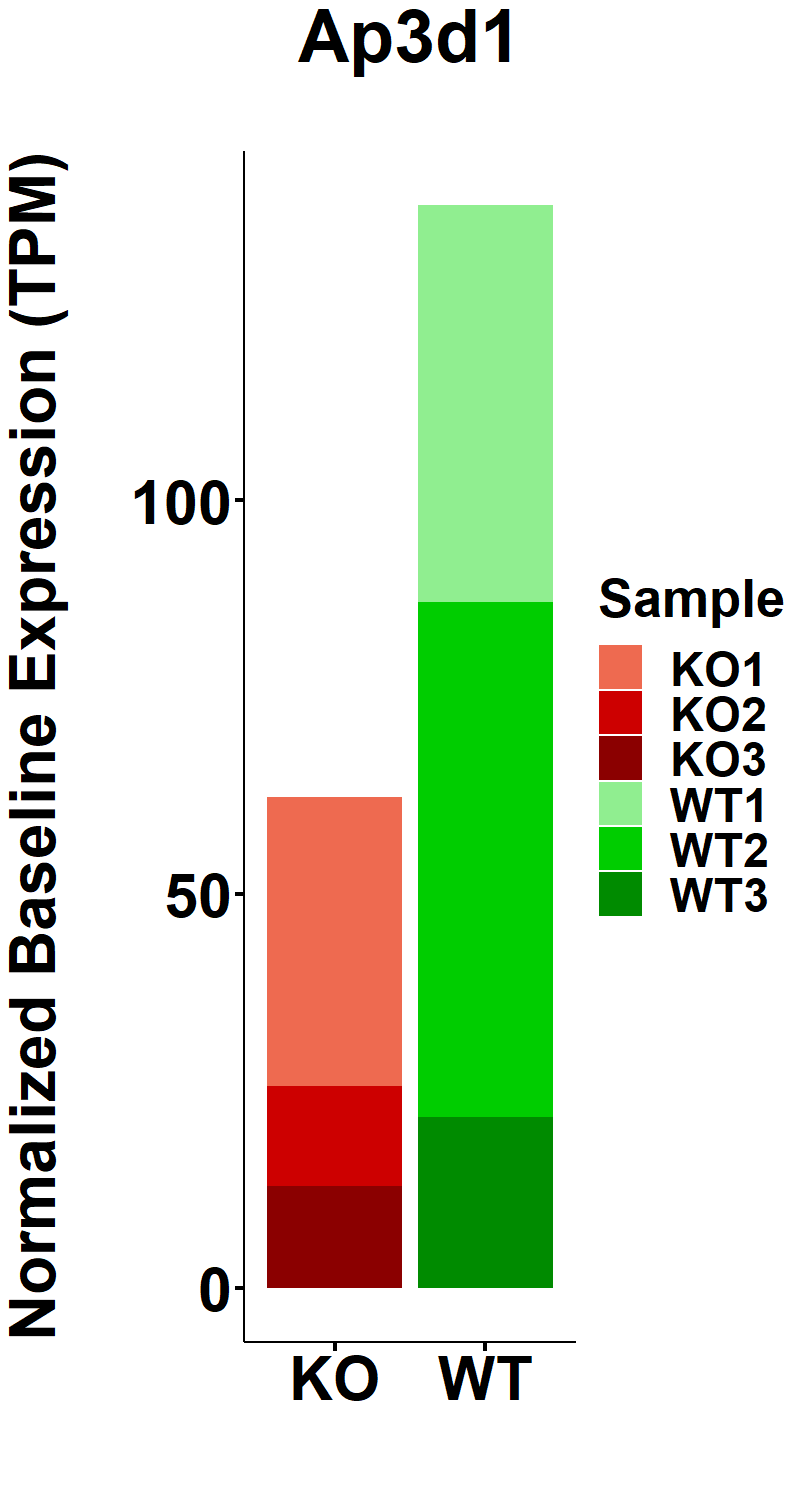** | **B**  **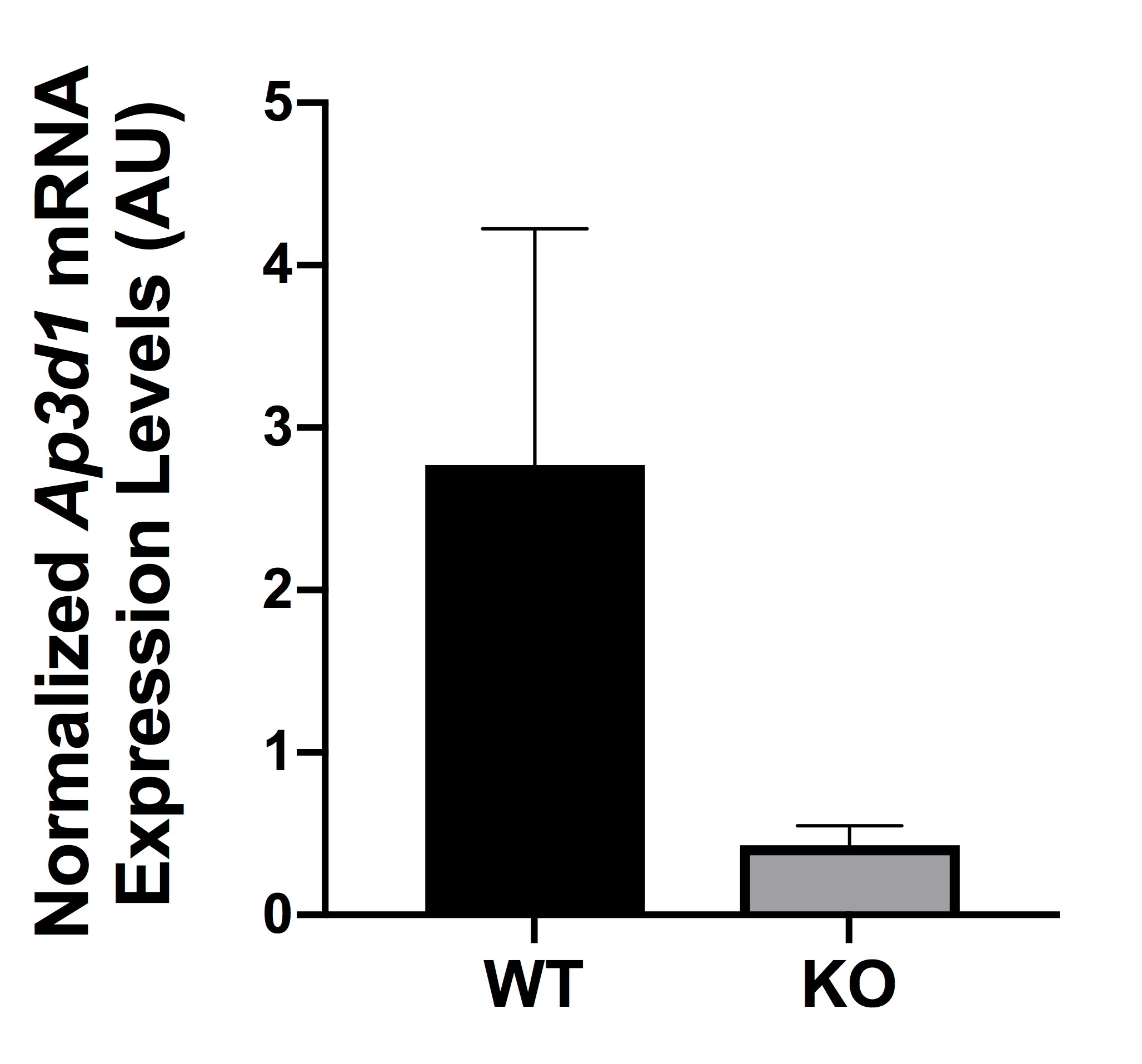** |
| --- | --- |

**Figure S2**. Baseline *Ap3d1* transcript levels of individual brain tissues taken from *Mcoln1^–/–^* knockout (KO1-KO3, n = 3) and *Mcoln1^+/+^* wild-type (WT1-WT3, n = 3) littermate control mice. **(A)** Transcriptomic and **(B)** real-time qPCR analyses of *Ap3d1* gene expression levels. The RNA-seq data were normalized using the TPM method. All qPCR experiments followed the MIQE guidelines. The qPCR experiments were done in triplicate wells, normalized using *18S rRNA*, and analyzed using the Standard Curve method. The qPCR data are represented as mean ± SD (n ≥ 3).

**REFERENCES**

1. Eichelsdoerfer JL, Evans JA, Slaugenhaupt SA, Cuajungco MP. Zinc dyshomeostasis is linked with the loss of mucolipidosis IV-associated TRPML1 ion channel. J Biol Chem. 2010;285(45):34304-8.

2. Bustin SA, Benes V, Garson JA, Hellemans J, Huggett J, Kubista M, et al. The MIQE guidelines: minimum information for publication of quantitative real-time PCR experiments. Clin Chem. 2009;55(4):611-22.

3. Venugopal B, Browning MF, Curcio-Morelli C, Varro A, Michaud N, Nanthakumar N, et al. Neurologic, gastric, and opthalmologic pathologies in a murine model of mucolipidosis type IV. Am J Hum Genet. 2007;81(5):1070-83.
