# Supplementary material for: ZnT3 expression levels are down-regulated in the brain of Mcoln1 knockout mice
Source: Mol Brain. 2019 Mar 26;12:24. doi: 10.1186/s13041-019-0446-3 (PMC6434829; doi:10.1186/s13041-019-0446-3)
Supplement: Supplementary file 2 — Table S3. Tabulated list of differentially expressed genes between Mcoln1−/− KO and Mcoln1+/+ WT control samples using DESeq2. (DOCX 150 kb) [file 13041_2019_446_MOESM2_ESM.docx]

**Additional file 2**

**ZnT3 expression levels are down-regulated in the brain of *Mcoln1* knockout mice**

**Jonathan Chacon, Lauren Rosas, and** **Math P. Cuajungco**

Department of Biological Science, and Center for Applied Biotechnology Studies, California State University Fullerton, Fullerton, CA, 92831, USA

**Corresponding author:**

Math P. Cuajungco, PhD

California State University Fullerton

Dept. of Biological Science

800 N. State College Blvd.

Fullerton, CA 92831

Tel: 657-278-8522

Fax: 657-278-3426

Jonathan Chacon, e-mail: jchacon27@csu.fullerton.edu

Lauren Rosas, e-mail: lrosas@csu.fullerton.edu

Math P. Cuajungco, e-mail: mcuajungco@fullerton.edu

**This file contains:**

Table S3

**Table S3.** Tabulated list of differentially expressed genes between *Mcoln1^–/–^* KO and *Mcoln1^+/+^* WT control samples using DESeq2. Both *Slc30a3* and *Mcoln1* genes relevant to the study are shown in bold text. The list contains significantly expressed genes (*p-value* < 0.05, Students *t*-test) and was sorted by fold-change using log2(FC) of down-regulated and up-regulated transcripts. Statistical analysis using Benjamani-Hochberg method for multiple hypothesis testing returned an adjusted p-value that was not significant for all listed genes. Validation using real-time qPCR and Western blot analyses were thus performed for *Slc30a3*.

| **Gene ID** | **Gene Symbol** | **Base mean** | **log2(FC)** | **Std Error** | **Wald-Stats** | **p-value** |
| --- | --- | --- | --- | --- | --- | --- |
| 73340 | nptxr | 248.715 | -1.488 | 0.388 | -3.833 | 0.000 |
| 384783 | IRS2 | 267.581 | -1.168 | 0.383 | -3.051 | 0.002 |
| 93708 | PCDHGC5 | 464.268 | -1.163 | 0.399 | -2.918 | 0.004 |
| 65079 | RTN4R | 259.990 | -1.157 | 0.391 | -2.957 | 0.003 |
| 75599 | PCDH1 | 666.783 | -1.151 | 0.419 | -2.744 | 0.006 |
| 381983 | lmtk3 | 493.944 | -1.138 | 0.387 | -2.943 | 0.003 |
| 12217 | BSN | 3782.978 | -1.136 | 0.471 | -2.411 | 0.016 |
| 381677 | VGF | 665.626 | -1.125 | 0.412 | -2.733 | 0.006 |
| 384569 | NOVA2 | 771.934 | -1.120 | 0.415 | -2.697 | 0.007 |
| 338521 | FA2H | 228.085 | -1.110 | 0.361 | -3.074 | 0.002 |
| 20665 | SOX10 | 201.780 | -1.108 | 0.354 | -3.128 | 0.002 |
| 17136 | MAG | 602.368 | -1.085 | 0.407 | -2.666 | 0.008 |
| 233878 | SEZ6L2 | 1223.962 | -1.083 | 0.437 | -2.477 | 0.013 |
| 13655 | EGR3 | 310.482 | -1.062 | 0.391 | -2.714 | 0.007 |
| 107448 | UNC5A | 438.050 | -1.062 | 0.391 | -2.718 | 0.007 |
| 13653 | EGR1 | 1531.526 | -1.060 | 0.461 | -2.299 | 0.021 |
| 232813 | SHISA7 | 699.717 | -1.049 | 0.395 | -2.657 | 0.008 |
| 546071 | MAST3 | 894.549 | -1.043 | 0.406 | -2.565 | 0.010 |
| 214240 | DISP2 | 1523.766 | -1.032 | 0.458 | -2.254 | 0.024 |
| 545276 | GAL3ST3 | 179.740 | -1.030 | 0.363 | -2.840 | 0.005 |
| 53883 | CELSR2 | 1282.053 | -1.029 | 0.444 | -2.316 | 0.021 |
| 50918 | MYADM | 214.504 | -1.025 | 0.377 | -2.718 | 0.007 |
| 78339 | TTYH3 | 675.436 | -1.024 | 0.413 | -2.478 | 0.013 |
| 16971 | LRP1 | 2516.665 | -1.012 | 0.444 | -2.277 | 0.023 |
| 269060 | DAGLA | 562.619 | -1.010 | 0.406 | -2.491 | 0.013 |
| 56747 | SEZ6L | 1109.839 | -1.002 | 0.431 | -2.328 | 0.020 |
| 243961 | SHANK1 | 5305.958 | -1.000 | 0.480 | -2.085 | 0.037 |
| 13656 | EGR4 | 98.861 | -0.999 | 0.372 | -2.689 | 0.007 |
| 20964 | SYN1 | 2981.060 | -0.999 | 0.473 | -2.112 | 0.035 |
| 16656 | HIVEP3 | 606.373 | -0.997 | 0.401 | -2.488 | 0.013 |
| 260297 | PRRT1 | 436.559 | -0.993 | 0.438 | -2.269 | 0.023 |
| 320587 | tmem88b | 259.978 | -0.993 | 0.373 | -2.663 | 0.008 |
| 319352 | PIANP | 601.691 | -0.993 | 0.424 | -2.343 | 0.019 |
| 244723 | OLFM2 | 312.395 | -0.992 | 0.384 | -2.581 | 0.010 |
| 216874 | CAMTA2 | 1012.070 | -0.989 | 0.430 | -2.299 | 0.022 |
| 20602 | NCOR2 | 1254.092 | -0.988 | 0.446 | -2.214 | 0.027 |
| 330319 | WIPF3 | 960.474 | -0.985 | 0.440 | -2.240 | 0.025 |
| 239102 | ZFHX2 | 431.947 | -0.985 | 0.373 | -2.644 | 0.008 |
| 18810 | PLEC | 1358.347 | -0.982 | 0.423 | -2.321 | 0.020 |
| 11305 | abca2 | 1302.979 | -0.982 | 0.422 | -2.326 | 0.020 |
| 14810 | GRIN1 | 1303.389 | -0.981 | 0.429 | -2.288 | 0.022 |
| 233210 | PRR12 | 547.459 | -0.978 | 0.430 | -2.273 | 0.023 |
| 73072 | PRR36 | 239.765 | -0.975 | 0.402 | -2.426 | 0.015 |
| 14104 | FASN | 1051.262 | -0.974 | 0.426 | -2.285 | 0.022 |
| 18752 | prkcg | 1027.909 | -0.967 | 0.428 | -2.261 | 0.024 |
| 13498 | ATN1 | 1432.210 | -0.962 | 0.457 | -2.107 | 0.035 |
| 108068 | GRM2 | 201.681 | -0.961 | 0.385 | -2.498 | 0.012 |
| 13429 | DNM1 | 3564.194 | -0.959 | 0.454 | -2.112 | 0.035 |
| 21375 | TBR1 | 615.021 | -0.957 | 0.405 | -2.360 | 0.018 |
| 68267 | SLC25A22 | 558.910 | -0.955 | 0.407 | -2.349 | 0.019 |
| 216856 | NLGN2 | 842.853 | -0.952 | 0.419 | -2.273 | 0.023 |
| 330814 | ADGRL1 | 2088.615 | -0.949 | 0.439 | -2.159 | 0.031 |
| 18830 | PLTP | 143.548 | -0.947 | 0.398 | -2.380 | 0.017 |
| 16549 | KHSRP | 336.921 | -0.946 | 0.373 | -2.540 | 0.011 |
| 71722 | CIC | 748.622 | -0.944 | 0.419 | -2.250 | 0.024 |
| 58996 | ARHGAP23 | 394.157 | -0.943 | 0.357 | -2.644 | 0.008 |
| 240057 | SYNGAP1 | 1332.757 | -0.943 | 0.418 | -2.258 | 0.024 |
| 53761 | PRRC2A | 1799.649 | -0.941 | 0.450 | -2.094 | 0.036 |
| 80749 | LRFN1 | 111.214 | -0.941 | 0.323 | -2.914 | 0.004 |
| 26562 | NCDN | 3000.785 | -0.939 | 0.454 | -2.067 | 0.039 |
| 74440 | CMIP | 1581.445 | -0.937 | 0.444 | -2.109 | 0.035 |
| 56013 | SRCIN1 | 1119.358 | -0.934 | 0.405 | -2.308 | 0.021 |
| 58234 | SHANK3 | 883.451 | -0.933 | 0.389 | -2.402 | 0.016 |
| 18032 | NFIX | 927.112 | -0.932 | 0.429 | -2.176 | 0.030 |
| 83674 | CNNM1 | 471.423 | -0.931 | 0.401 | -2.320 | 0.020 |
| 235431 | CORO2B | 756.870 | -0.930 | 0.418 | -2.222 | 0.026 |
| 65945 | CLSTN1 | 2946.001 | -0.930 | 0.456 | -2.038 | 0.042 |
| 381353 | Gm996 | 341.797 | -0.929 | 0.363 | -2.561 | 0.010 |
| 20660 | SORL1 | 1305.686 | -0.927 | 0.429 | -2.160 | 0.031 |
| 19679 | PITPNM2 | 1109.100 | -0.926 | 0.411 | -2.254 | 0.024 |
| 230775 | ADGRB2 | 1153.129 | -0.926 | 0.405 | -2.286 | 0.022 |
| 23805 | APC2 | 687.648 | -0.924 | 0.396 | -2.331 | 0.020 |
| 233071 | arhgap33 | 537.925 | -0.923 | 0.382 | -2.420 | 0.016 |
| 269295 | RTN4RL2 | 67.063 | -0.922 | 0.411 | -2.244 | 0.025 |
| 110891 | SLC8A2 | 903.112 | -0.922 | 0.416 | -2.217 | 0.027 |
| 68846 | RNF208 | 246.681 | -0.918 | 0.387 | -2.371 | 0.018 |
| 17261 | MEF2D | 825.227 | -0.917 | 0.412 | -2.229 | 0.026 |
| 107939 | pom121 | 483.076 | -0.916 | 0.433 | -2.114 | 0.034 |
| 20312 | CX3CL1 | 2335.697 | -0.915 | 0.444 | -2.061 | 0.039 |
| 20222 | SF3A2 | 153.991 | -0.913 | 0.375 | -2.438 | 0.015 |
| 57138 | SLC12A5 | 1523.358 | -0.913 | 0.435 | -2.099 | 0.036 |
| 381022 | KMT2D | 1496.574 | -0.913 | 0.426 | -2.144 | 0.032 |
| 320878 | MICAL2 | 1139.732 | -0.912 | 0.406 | -2.247 | 0.025 |
| 27801 | ZDHHC8 | 483.959 | -0.912 | 0.409 | -2.230 | 0.026 |
| 223693 | TMEM184B | 323.623 | -0.907 | 0.369 | -2.456 | 0.014 |
| 382034 | GSE1 | 476.450 | -0.904 | 0.398 | -2.273 | 0.023 |
| 545554 | ANKRD34A | 271.834 | -0.903 | 0.385 | -2.344 | 0.019 |
| 435965 | LRP3 | 221.553 | -0.900 | 0.335 | -2.683 | 0.007 |
| 13424 | DYNC1H1 | 3907.340 | -0.899 | 0.453 | -1.983 | 0.047 |
| 269700 | Gm15800 | 2213.922 | -0.898 | 0.428 | -2.097 | 0.036 |
| 13191 | DCTN1 | 942.070 | -0.895 | 0.405 | -2.213 | 0.027 |
| 75607 | WNK2 | 705.499 | -0.895 | 0.405 | -2.211 | 0.027 |
| 104027 | SYNPO | 701.285 | -0.894 | 0.394 | -2.270 | 0.023 |
| 20544 | SLC9A1 | 262.397 | -0.894 | 0.388 | -2.304 | 0.021 |
| 100213 | RUSC2 | 610.241 | -0.893 | 0.391 | -2.283 | 0.022 |
| 320365 | FRY | 1425.807 | -0.893 | 0.424 | -2.105 | 0.035 |
| 216439 | AGAP2 | 2839.779 | -0.891 | 0.438 | -2.034 | 0.042 |
| 27967 | CHERP | 331.274 | -0.890 | 0.379 | -2.349 | 0.019 |
| 226180 | INA | 535.492 | -0.890 | 0.419 | -2.121 | 0.034 |
| 14387 | gaa | 1188.522 | -0.888 | 0.437 | -2.034 | 0.042 |
| 14086 | FSCN1 | 667.213 | -0.887 | 0.399 | -2.224 | 0.026 |
| 19275 | PTPRN | 1747.037 | -0.886 | 0.429 | -2.064 | 0.039 |
| 18164 | NPTX1 | 1201.314 | -0.885 | 0.431 | -2.053 | 0.040 |
| 50794 | KLF13 | 688.501 | -0.884 | 0.389 | -2.273 | 0.023 |
| 192159 | PRPF8 | 1253.156 | -0.883 | 0.424 | -2.084 | 0.037 |
| 18626 | PER1 | 625.426 | -0.882 | 0.431 | -2.046 | 0.041 |
| 235402 | LINGO1 | 1251.229 | -0.882 | 0.433 | -2.036 | 0.042 |
| 241727 | SNPH | 1209.913 | -0.880 | 0.425 | -2.070 | 0.038 |
| 225908 | MYRF | 341.216 | -0.878 | 0.393 | -2.236 | 0.025 |
| 18218 | DUSP8 | 453.718 | -0.878 | 0.387 | -2.270 | 0.023 |
| 18029 | nfic | 722.073 | -0.878 | 0.415 | -2.114 | 0.035 |
| 64652 | NISCH | 1801.994 | -0.877 | 0.437 | -2.005 | 0.045 |
| 328365 | ZMIZ1 | 1114.520 | -0.877 | 0.438 | -1.999 | 0.046 |
| 11975 | ATP6V0A1 | 1588.769 | -0.874 | 0.426 | -2.050 | 0.040 |
| 71901 | FAM219A | 261.631 | -0.874 | 0.351 | -2.489 | 0.013 |
| 244058 | RGMA | 246.738 | -0.874 | 0.368 | -2.372 | 0.018 |
| 11771 | AP2A1 | 760.273 | -0.871 | 0.402 | -2.170 | 0.030 |
| 245860 | atg9a | 338.578 | -0.871 | 0.371 | -2.349 | 0.019 |
| 233919 | GPR26 | 229.418 | -0.871 | 0.337 | -2.582 | 0.010 |
| 270058 | MAP1S | 205.053 | -0.869 | 0.353 | -2.463 | 0.014 |
| 80297 | SPTBN4 | 574.734 | -0.869 | 0.354 | -2.456 | 0.014 |
| 319984 | JPH4 | 1379.261 | -0.868 | 0.418 | -2.078 | 0.038 |
| 99296 | HRH3 | 195.257 | -0.868 | 0.351 | -2.475 | 0.013 |
| 83767 | wasf1 | 771.673 | -0.868 | 0.424 | -2.045 | 0.041 |
| 72097 | 2010300C02Rik | 719.950 | -0.867 | 0.396 | -2.188 | 0.029 |
| 227723 | PRRC2B | 2502.359 | -0.867 | 0.441 | -1.966 | 0.049 |
| 22393 | WFS1 | 328.319 | -0.867 | 0.383 | -2.264 | 0.024 |
| 16512 | kcnh3 | 354.826 | -0.866 | 0.349 | -2.478 | 0.013 |
| 57261 | BRD4 | 799.211 | -0.866 | 0.417 | -2.076 | 0.038 |
| 52915 | ZMIZ2 | 1233.683 | -0.865 | 0.415 | -2.085 | 0.037 |
| 235044 | PLPPR2 | 441.804 | -0.861 | 0.400 | -2.153 | 0.031 |
| 217166 | NR1D1 | 885.181 | -0.860 | 0.438 | -1.963 | 0.050 |
| 117160 | TTYH2 | 225.908 | -0.860 | 0.345 | -2.495 | 0.013 |
| 207393 | ELFN2 | 526.787 | -0.860 | 0.388 | -2.214 | 0.027 |
| 606496 | GSK3A | 423.288 | -0.859 | 0.388 | -2.214 | 0.027 |
| 227580 | C1QL3 | 347.805 | -0.859 | 0.395 | -2.175 | 0.030 |
| 56381 | SPEN | 875.921 | -0.859 | 0.400 | -2.149 | 0.032 |
| 20370 | SEZ6 | 1063.316 | -0.858 | 0.422 | -2.032 | 0.042 |
| 13116 | cyp46a1 | 670.494 | -0.858 | 0.403 | -2.130 | 0.033 |
| 330790 | HAPLN4 | 403.818 | -0.857 | 0.408 | -2.100 | 0.036 |
| 12669 | CHRM1 | 727.565 | -0.857 | 0.413 | -2.076 | 0.038 |
| 269713 | CLIP2 | 472.854 | -0.857 | 0.362 | -2.368 | 0.018 |
| 231876 | LMTK2 | 1112.358 | -0.857 | 0.415 | -2.065 | 0.039 |
| 11302 | AATK | 838.435 | -0.855 | 0.401 | -2.131 | 0.033 |
| 238330 | IRF2BPL | 267.004 | -0.855 | 0.368 | -2.324 | 0.020 |
| 75687 | FAM65A | 500.476 | -0.854 | 0.402 | -2.126 | 0.033 |
| 233208 | SCAF1 | 665.082 | -0.854 | 0.398 | -2.146 | 0.032 |
| 11838 | ARC | 659.707 | -0.854 | 0.401 | -2.128 | 0.033 |
| 382056 | CRTC1 | 683.113 | -0.853 | 0.388 | -2.195 | 0.028 |
| 22084 | TSC2 | 499.390 | -0.852 | 0.381 | -2.239 | 0.025 |
| 319586 | CELF5 | 1199.315 | -0.852 | 0.407 | -2.092 | 0.036 |
| 382018 | UNC13A | 1277.780 | -0.852 | 0.412 | -2.066 | 0.039 |
| 20020 | POLR2A | 600.535 | -0.852 | 0.422 | -2.018 | 0.044 |
| 59090 | MIDN | 369.936 | -0.851 | 0.380 | -2.241 | 0.025 |
| 20893 | BHLHE40 | 454.214 | -0.851 | 0.419 | -2.031 | 0.042 |
| 29813 | Zfp385a | 343.689 | -0.851 | 0.390 | -2.179 | 0.029 |
| 100043597 | SRCAP | 1171.145 | -0.850 | 0.413 | -2.057 | 0.040 |
| 242773 | SLC45A1 | 153.787 | -0.849 | 0.347 | -2.450 | 0.014 |
| 231440 | PARM1 | 201.317 | -0.849 | 0.364 | -2.333 | 0.020 |
| 330369 | FBXO41 | 437.101 | -0.847 | 0.364 | -2.330 | 0.020 |
| 243931 | TSHZ3 | 285.166 | -0.847 | 0.359 | -2.357 | 0.018 |
| 208043 | SETD1B | 416.645 | -0.846 | 0.403 | -2.100 | 0.036 |
| 245666 | IQSEC2 | 995.514 | -0.846 | 0.418 | -2.022 | 0.043 |
| 216971 | FAM222B | 291.781 | -0.842 | 0.368 | -2.286 | 0.022 |
| 239556 | CACNA1I | 518.692 | -0.842 | 0.382 | -2.201 | 0.028 |
| 56417 | ADAR | 795.845 | -0.841 | 0.407 | -2.069 | 0.039 |
| 13138 | DAG1 | 547.129 | -0.840 | 0.408 | -2.059 | 0.040 |
| 57778 | FMNL1 | 465.510 | -0.838 | 0.381 | -2.197 | 0.028 |
| 208884 | ZDHHC9 | 224.718 | -0.837 | 0.361 | -2.319 | 0.020 |
| 11881 | ARSB | 630.498 | -0.836 | 0.419 | -1.995 | 0.046 |
| 217882 | CEP170B | 1277.156 | -0.835 | 0.420 | -1.989 | 0.047 |
| 223604 | KCNK9 | 62.310 | -0.835 | 0.373 | -2.240 | 0.025 |
| 70729 | NOS1AP | 277.608 | -0.835 | 0.344 | -2.427 | 0.015 |
| 16500 | KCNB1 | 1505.797 | -0.834 | 0.422 | -1.977 | 0.048 |
| 107831 | ADGRB1 | 1425.208 | -0.834 | 0.415 | -2.007 | 0.045 |
| 99738 | KCNC4 | 243.766 | -0.833 | 0.359 | -2.321 | 0.020 |
| 232976 | Zfp574 | 172.755 | -0.831 | 0.322 | -2.583 | 0.010 |
| 93706 | PCDHGC3 | 269.862 | -0.831 | 0.356 | -2.336 | 0.019 |
| 230082 | NOL6 | 408.738 | -0.831 | 0.388 | -2.144 | 0.032 |
| 70435 | INF2 | 520.249 | -0.830 | 0.365 | -2.273 | 0.023 |
| 109349 | FAM163B | 572.966 | -0.830 | 0.422 | -1.964 | 0.050 |
| 56527 | MAST1 | 404.066 | -0.829 | 0.376 | -2.206 | 0.027 |
| 20239 | ATXN2 | 726.193 | -0.827 | 0.398 | -2.078 | 0.038 |
| 106763 | TTBK1 | 458.709 | -0.827 | 0.359 | -2.301 | 0.021 |
| 230793 | AHDC1 | 388.160 | -0.826 | 0.383 | -2.156 | 0.031 |
| 353310 | Zfp703 | 214.355 | -0.824 | 0.374 | -2.204 | 0.028 |
| 14763 | GPR37 | 227.194 | -0.822 | 0.356 | -2.308 | 0.021 |
| 73001 | 2900055J20Rik | 76.149 | -0.822 | 0.403 | -2.037 | 0.042 |
| 268480 | RAPGEFL1 | 422.430 | -0.821 | 0.366 | -2.245 | 0.025 |
| 18577 | PDE4A | 521.865 | -0.820 | 0.385 | -2.132 | 0.033 |
| 232370 | clstn3 | 841.447 | -0.820 | 0.412 | -1.993 | 0.046 |
| 170729 | SCRT1 | 715.390 | -0.819 | 0.391 | -2.096 | 0.036 |
| 16502 | KCNC1 | 944.163 | -0.818 | 0.413 | -1.983 | 0.047 |
| 77582 | MBOAT7 | 380.258 | -0.818 | 0.383 | -2.138 | 0.033 |
| 16728 | L1CAM | 715.384 | -0.815 | 0.393 | -2.075 | 0.038 |
| 20340 | GLG1 | 532.032 | -0.815 | 0.390 | -2.087 | 0.037 |
| 19377 | RAI1 | 476.368 | -0.811 | 0.368 | -2.202 | 0.028 |
| 224727 | BAG6 | 667.416 | -0.810 | 0.391 | -2.072 | 0.038 |
| 80909 | GATSL2 | 458.730 | -0.809 | 0.374 | -2.162 | 0.031 |
| 16980 | LRRN2 | 426.963 | -0.808 | 0.396 | -2.041 | 0.041 |
| 12672 | CHRM4 | 149.338 | -0.807 | 0.340 | -2.377 | 0.017 |
| 81907 | TMEM108 | 134.507 | -0.807 | 0.319 | -2.531 | 0.011 |
| 100732 | MAPRE3 | 626.400 | -0.806 | 0.388 | -2.079 | 0.038 |
| 15370 | NR4A1 | 334.125 | -0.806 | 0.375 | -2.148 | 0.032 |
| 381338 | LONRF2 | 760.757 | -0.805 | 0.411 | -1.962 | 0.050 |
| 99010 | LPCAT4 | 386.827 | -0.805 | 0.384 | -2.094 | 0.036 |
| 71772 | PLBD2 | 276.728 | -0.803 | 0.375 | -2.139 | 0.032 |
| 23964 | TENM2 | 961.243 | -0.802 | 0.408 | -1.965 | 0.049 |
| 12286 | CACNA1A | 1049.407 | -0.801 | 0.400 | -2.006 | 0.045 |
| 18844 | plxna1 | 560.515 | -0.801 | 0.395 | -2.027 | 0.043 |
| 227693 | ZER1 | 623.958 | -0.801 | 0.388 | -2.064 | 0.039 |
| 59033 | SLC4A8 | 783.964 | -0.800 | 0.400 | -2.002 | 0.045 |
| 12295 | CACNB1 | 331.794 | -0.800 | 0.356 | -2.249 | 0.024 |
| 104156 | ETV5 | 341.411 | -0.799 | 0.349 | -2.290 | 0.022 |
| 58235 | NECTIN1 | 334.216 | -0.796 | 0.389 | -2.045 | 0.041 |
| 214597 | SIDT2 | 232.892 | -0.796 | 0.318 | -2.504 | 0.012 |
| 235320 | ZBTB16 | 293.287 | -0.795 | 0.364 | -2.186 | 0.029 |
| 20562 | SLIT1 | 383.276 | -0.795 | 0.366 | -2.170 | 0.030 |
| 233020 | HIPK4 | 156.758 | -0.794 | 0.322 | -2.467 | 0.014 |
| 72296 | RUSC1 | 469.085 | -0.794 | 0.384 | -2.064 | 0.039 |
| 13688 | EIF4EBP2 | 146.535 | -0.793 | 0.337 | -2.350 | 0.019 |
| 11603 | AGRN | 458.055 | -0.792 | 0.338 | -2.340 | 0.019 |
| 269389 | TOX2 | 130.327 | -0.791 | 0.373 | -2.120 | 0.034 |
| 231861 | TNRC18 | 590.175 | -0.791 | 0.397 | -1.992 | 0.046 |
| 105239 | RNF44 | 703.660 | -0.790 | 0.400 | -1.974 | 0.048 |
| 228355 | MADD | 787.651 | -0.790 | 0.396 | -1.996 | 0.046 |
| 78779 | spata2l | 148.253 | -0.789 | 0.329 | -2.398 | 0.017 |
| 15228 | FOXG1 | 581.040 | -0.787 | 0.378 | -2.082 | 0.037 |
| 27410 | ABCA3 | 351.391 | -0.786 | 0.370 | -2.123 | 0.034 |
| 14809 | GRIK5 | 714.393 | -0.782 | 0.386 | -2.026 | 0.043 |
| 14811 | GRIN2A | 512.428 | -0.782 | 0.390 | -2.006 | 0.045 |
| 54376 | CACNG3 | 391.069 | -0.782 | 0.389 | -2.009 | 0.045 |
| 140559 | IGSF8 | 370.409 | -0.780 | 0.387 | -2.013 | 0.044 |
| 210274 | SHANK2 | 874.258 | -0.779 | 0.387 | -2.012 | 0.044 |
| 269878 | MEGF8 | 483.457 | -0.778 | 0.369 | -2.110 | 0.035 |
| 18530 | PCDH8 | 108.594 | -0.778 | 0.362 | -2.149 | 0.032 |
| 237615 | ANKRD52 | 372.785 | -0.777 | 0.369 | -2.106 | 0.035 |
| 243842 | GLTSCR1 | 272.779 | -0.777 | 0.364 | -2.132 | 0.033 |
| 75613 | MED25 | 301.627 | -0.776 | 0.352 | -2.207 | 0.027 |
| 68051 | NUTF2 | 90.651 | -0.776 | 0.342 | -2.266 | 0.023 |
| 268721 | ZSWIM8 | 501.874 | -0.775 | 0.362 | -2.139 | 0.032 |
| 68799 | RGMB | 200.891 | -0.773 | 0.351 | -2.204 | 0.028 |
| 66961 | NEAT1 | 265.697 | -0.773 | 0.393 | -1.965 | 0.049 |
| 380702 | SHISA6 | 263.182 | -0.773 | 0.392 | -1.969 | 0.049 |
| **22784** | **SLC30A3** | **181.010** | **-0.772** | **0.333** | **-2.317** | **0.021** |
| 76992 | Fam219aos | 126.460 | -0.771 | 0.359 | -2.150 | 0.032 |
| 19719 | RFNG | 272.059 | -0.770 | 0.370 | -2.081 | 0.037 |
| 72043 | SULF2 | 508.491 | -0.769 | 0.382 | -2.011 | 0.044 |
| 11550 | ADRA1D | 90.393 | -0.767 | 0.316 | -2.427 | 0.015 |
| 212712 | SATB2 | 468.477 | -0.766 | 0.376 | -2.036 | 0.042 |
| 76484 | KNDC1 | 688.712 | -0.764 | 0.389 | -1.963 | 0.050 |
| 22761 | ZFPM1 | 187.360 | -0.764 | 0.334 | -2.286 | 0.022 |
| 80906 | kcnip2 | 100.417 | -0.764 | 0.317 | -2.406 | 0.016 |
| 142682 | ZCCHC14 | 424.683 | -0.764 | 0.361 | -2.112 | 0.035 |
| 21367 | CNTN2 | 491.461 | -0.763 | 0.366 | -2.086 | 0.037 |
| 81905 | CACNG8 | 160.767 | -0.762 | 0.349 | -2.183 | 0.029 |
| 55979 | AGPAT1 | 413.756 | -0.762 | 0.379 | -2.013 | 0.044 |
| 16565 | KIF21B | 508.791 | -0.756 | 0.350 | -2.161 | 0.031 |
| 99237 | TM9SF4 | 382.386 | -0.756 | 0.366 | -2.069 | 0.039 |
| 244310 | DLGAP2 | 469.554 | -0.756 | 0.363 | -2.084 | 0.037 |
| 13643 | EFNB3 | 280.143 | -0.754 | 0.367 | -2.054 | 0.040 |
| 104401 | PCNX3 | 274.930 | -0.754 | 0.367 | -2.056 | 0.040 |
| 319998 | TMEM198 | 150.418 | -0.754 | 0.314 | -2.399 | 0.016 |
| 98432 | PHLPP1 | 497.637 | -0.753 | 0.373 | -2.019 | 0.044 |
| 74123 | FOXP4 | 240.154 | -0.753 | 0.362 | -2.083 | 0.037 |
| 98170 | tmem132a | 419.055 | -0.751 | 0.382 | -1.965 | 0.049 |
| 13544 | DVL3 | 271.201 | -0.749 | 0.359 | -2.088 | 0.037 |
| 268510 | MGAT5B | 283.571 | -0.749 | 0.335 | -2.235 | 0.025 |
| 224807 | TMEM63B | 456.229 | -0.748 | 0.370 | -2.020 | 0.043 |
| 80288 | BCL9L | 257.167 | -0.747 | 0.356 | -2.101 | 0.036 |
| 74754 | DHCR24 | 407.909 | -0.745 | 0.373 | -1.998 | 0.046 |
| 74126 | SYVN1 | 218.427 | -0.745 | 0.360 | -2.067 | 0.039 |
| 20014 | RPN2 | 217.068 | -0.743 | 0.341 | -2.177 | 0.029 |
| 13549 | DYRK1B | 196.901 | -0.742 | 0.303 | -2.449 | 0.014 |
| 18223 | NUMBL | 325.452 | -0.742 | 0.358 | -2.073 | 0.038 |
| 78455 | HELZ | 449.685 | -0.740 | 0.355 | -2.083 | 0.037 |
| 20564 | SLIT3 | 286.352 | -0.740 | 0.339 | -2.181 | 0.029 |
| 71764 | C2CD2L | 513.031 | -0.739 | 0.368 | -2.008 | 0.045 |
| 22793 | ZYX | 186.647 | -0.739 | 0.361 | -2.045 | 0.041 |
| 232791 | CNOT3 | 210.021 | -0.739 | 0.344 | -2.147 | 0.032 |
| 17294 | MEST | 316.233 | -0.738 | 0.349 | -2.116 | 0.034 |
| 11515 | ADCY9 | 299.771 | -0.737 | 0.355 | -2.074 | 0.038 |
| 68490 | Zfp579 | 65.579 | -0.736 | 0.345 | -2.135 | 0.033 |
| 71947 | TMEM94 | 268.050 | -0.735 | 0.358 | -2.051 | 0.040 |
| 224129 | ADCY5 | 623.582 | -0.735 | 0.354 | -2.078 | 0.038 |
| 263876 | SPATA2 | 208.299 | -0.735 | 0.352 | -2.084 | 0.037 |
| 241794 | KCNG1 | 82.547 | -0.735 | 0.347 | -2.117 | 0.034 |
| 217219 | FAM171A2 | 198.851 | -0.733 | 0.330 | -2.223 | 0.026 |
| 12305 | ddr1 | 205.178 | -0.732 | 0.315 | -2.323 | 0.020 |
| 239528 | AGO2 | 638.601 | -0.732 | 0.364 | -2.011 | 0.044 |
| 71766 | RAVER1 | 217.680 | -0.731 | 0.339 | -2.157 | 0.031 |
| 383348 | KCTD16 | 124.769 | -0.731 | 0.333 | -2.193 | 0.028 |
| 70638 | FAM189A1 | 331.363 | -0.731 | 0.356 | -2.051 | 0.040 |
| 50817 | CAPN15 | 73.632 | -0.730 | 0.325 | -2.242 | 0.025 |
| 103406 | ZFR2 | 178.343 | -0.729 | 0.306 | -2.382 | 0.017 |
| 77630 | PRDM8 | 170.092 | -0.728 | 0.335 | -2.173 | 0.030 |
| 110058 | syt17 | 123.162 | -0.727 | 0.317 | -2.298 | 0.022 |
| 195646 | HS3ST2 | 125.807 | -0.725 | 0.304 | -2.382 | 0.017 |
| 12053 | BCL6 | 137.980 | -0.725 | 0.365 | -1.987 | 0.047 |
| 232879 | ZBTB45 | 86.975 | -0.725 | 0.336 | -2.158 | 0.031 |
| 623954 | Gm10493 | 150.953 | -0.725 | 0.360 | -2.013 | 0.044 |
| 22404 | WIZ | 227.043 | -0.724 | 0.367 | -1.972 | 0.049 |
| 13446 | DOC2A | 154.112 | -0.724 | 0.324 | -2.233 | 0.026 |
| 215335 | SLC36A1 | 259.649 | -0.721 | 0.331 | -2.177 | 0.029 |
| 116870 | MTA1 | 257.185 | -0.718 | 0.333 | -2.158 | 0.031 |
| 215445 | RAB11FIP3 | 422.064 | -0.718 | 0.356 | -2.015 | 0.044 |
| 24045 | SCAMP3 | 218.301 | -0.714 | 0.334 | -2.141 | 0.032 |
| 68617 | MTCL1 | 531.114 | -0.713 | 0.355 | -2.010 | 0.044 |
| 98366 | SMAP1 | 275.337 | -0.711 | 0.363 | -1.961 | 0.050 |
| 109711 | ACTN1 | 363.046 | -0.710 | 0.326 | -2.177 | 0.030 |
| 432763 | PRR7 | 108.636 | -0.709 | 0.321 | -2.207 | 0.027 |
| 76893 | CERS2 | 161.201 | -0.708 | 0.322 | -2.198 | 0.028 |
| 73750 | WHRN | 216.211 | -0.705 | 0.319 | -2.209 | 0.027 |
| 11488 | ADAM11 | 385.502 | -0.704 | 0.352 | -1.998 | 0.046 |
| 433938 | MN1 | 297.319 | -0.704 | 0.350 | -2.011 | 0.044 |
| 242409 | TMEM8B | 257.182 | -0.702 | 0.335 | -2.097 | 0.036 |
| 224897 | DPP9 | 245.956 | -0.701 | 0.352 | -1.995 | 0.046 |
| 105439 | SLAIN1 | 242.679 | -0.700 | 0.346 | -2.019 | 0.043 |
| 269037 | CTIF | 343.662 | -0.698 | 0.356 | -1.961 | 0.050 |
| 12287 | CACNA1B | 569.424 | -0.696 | 0.350 | -1.987 | 0.047 |
| 214063 | DNAJC16 | 239.679 | -0.692 | 0.335 | -2.067 | 0.039 |
| 232944 | MARK4 | 236.127 | -0.692 | 0.342 | -2.026 | 0.043 |
| 108657 | RNPEPL1 | 100.747 | -0.692 | 0.290 | -2.382 | 0.017 |
| 76220 | 6530402F18Rik | 127.831 | -0.691 | 0.296 | -2.339 | 0.019 |
| 268448 | PHF12 | 283.217 | -0.691 | 0.344 | -2.010 | 0.044 |
| 14123 | FBRS | 228.583 | -0.688 | 0.340 | -2.025 | 0.043 |
| 93715 | PCDHGA7 | 122.880 | -0.688 | 0.334 | -2.060 | 0.039 |
| 53860 | BOD1 | 268.291 | -0.686 | 0.336 | -2.044 | 0.041 |
| 17690 | MSI1 | 129.027 | -0.686 | 0.339 | -2.025 | 0.043 |
| 26457 | SLC27A1 | 179.843 | -0.685 | 0.335 | -2.045 | 0.041 |
| 381126 | GAREM1 | 245.834 | -0.683 | 0.320 | -2.133 | 0.033 |
| **94178** | **MCOLN1** | **169.956** | **-0.682** | **0.322** | **-2.115** | **0.034** |
| 235086 | IGSF9B | 239.184 | -0.678 | 0.307 | -2.207 | 0.027 |
| 625638 | FAM43B | 72.584 | -0.677 | 0.335 | -2.022 | 0.043 |
| 329540 | NOL4L | 387.679 | -0.674 | 0.340 | -1.981 | 0.048 |
| 114873 | dscaml1 | 334.957 | -0.671 | 0.338 | -1.987 | 0.047 |
| 53607 | SNRPA | 138.950 | -0.669 | 0.341 | -1.961 | 0.050 |
| 230796 | WDTC1 | 289.093 | -0.666 | 0.323 | -2.062 | 0.039 |
| 19089 | PRKCSH | 202.595 | -0.665 | 0.339 | -1.962 | 0.050 |
| 53324 | NPTX2 | 150.053 | -0.663 | 0.324 | -2.044 | 0.041 |
| 67789 | DALRD3 | 138.403 | -0.659 | 0.316 | -2.084 | 0.037 |
| 110962 | mbd6 | 214.096 | -0.658 | 0.310 | -2.123 | 0.034 |
| 78885 | CORO7 | 228.278 | -0.653 | 0.329 | -1.987 | 0.047 |
| 231863 | FBXL18 | 124.699 | -0.647 | 0.293 | -2.208 | 0.027 |
| 238384 | SLC24A4 | 103.263 | -0.645 | 0.284 | -2.272 | 0.023 |
| 233781 | XYLT1 | 167.567 | -0.641 | 0.295 | -2.171 | 0.030 |
| 224705 | VPS52 | 191.542 | -0.641 | 0.316 | -2.024 | 0.043 |
| 260302 | GGA3 | 245.519 | -0.638 | 0.317 | -2.016 | 0.044 |
| 330485 | TMEM145 | 130.779 | -0.637 | 0.312 | -2.038 | 0.042 |
| 171171 | NTNG2 | 88.774 | -0.635 | 0.309 | -2.052 | 0.040 |
| 71780 | ISYNA1 | 110.209 | -0.634 | 0.311 | -2.042 | 0.041 |
| 14073 | FAAH | 189.539 | -0.628 | 0.312 | -2.013 | 0.044 |
| 229722 | 5330417C22Rik | 167.207 | -0.626 | 0.289 | -2.164 | 0.030 |
| 53627 | porcn | 141.717 | -0.620 | 0.315 | -1.971 | 0.049 |
| 228536 | BAHD1 | 172.467 | -0.618 | 0.313 | -1.975 | 0.048 |
| 215303 | CAMK1G | 145.538 | -0.611 | 0.310 | -1.969 | 0.049 |
| 17155 | Man1a | 181.164 | -0.608 | 0.309 | -1.969 | 0.049 |
| 107305 | VPS37C | 89.633 | -0.602 | 0.304 | -1.977 | 0.048 |
| 93716 | PCDHGA8 | 93.627 | -0.570 | 0.290 | -1.966 | 0.049 |
| 72345 | AMER1 | 146.664 | 0.580 | 0.293 | 1.980 | 0.048 |
| 213391 | RASSF4 | 120.998 | 0.589 | 0.294 | 2.004 | 0.045 |
| 245174 | Zfp937 | 104.152 | 0.618 | 0.294 | 2.099 | 0.036 |
| 72776 | SASS6 | 86.595 | 0.627 | 0.311 | 2.014 | 0.044 |
| 71586 | IFIH1 | 101.766 | 0.630 | 0.316 | 1.994 | 0.046 |
| 209086 | SAMD9L | 102.225 | 0.634 | 0.287 | 2.207 | 0.027 |
| 56193 | PLEK | 124.684 | 0.636 | 0.298 | 2.135 | 0.033 |
| 320226 | CCDC171 | 108.015 | 0.636 | 0.292 | 2.177 | 0.029 |
| 245368 | Zfp300 | 74.122 | 0.642 | 0.326 | 1.969 | 0.049 |
| 67071 | RPS6KA6 | 91.957 | 0.644 | 0.321 | 2.007 | 0.045 |
| 242509 | BNC2 | 99.341 | 0.653 | 0.327 | 1.997 | 0.046 |
| 210009 | MTRR | 86.772 | 0.654 | 0.317 | 2.067 | 0.039 |
| 320965 | 4831440E17Rik | 65.297 | 0.661 | 0.325 | 2.036 | 0.042 |
| 54486 | HPGDS | 67.427 | 0.661 | 0.326 | 2.028 | 0.043 |
| 574403 | FAM196B | 80.151 | 0.664 | 0.305 | 2.175 | 0.030 |
| 108154 | ADAMTS6 | 101.336 | 0.680 | 0.316 | 2.154 | 0.031 |
| 24000 | PTPN21 | 117.645 | 0.682 | 0.294 | 2.318 | 0.020 |
| 229900 | gbp7 | 98.791 | 0.682 | 0.297 | 2.294 | 0.022 |
| 16170 | IL16 | 91.871 | 0.686 | 0.345 | 1.991 | 0.046 |
| 12816 | COL12A1 | 202.871 | 0.690 | 0.345 | 2.000 | 0.046 |
| 66412 | ARRDC4 | 63.629 | 0.692 | 0.328 | 2.111 | 0.035 |
| 217262 | ABCA9 | 124.653 | 0.697 | 0.298 | 2.340 | 0.019 |
| 80859 | nfkbiz | 68.434 | 0.700 | 0.329 | 2.124 | 0.034 |
| 19696 | REL | 53.447 | 0.704 | 0.353 | 1.994 | 0.046 |
| 78781 | ZC3HAV1 | 76.516 | 0.705 | 0.339 | 2.077 | 0.038 |
| 16425 | ITIH2 | 70.097 | 0.708 | 0.358 | 1.976 | 0.048 |
| 14211 | SMC2 | 126.354 | 0.710 | 0.288 | 2.467 | 0.014 |
| 624866 | LEKR1 | 75.339 | 0.712 | 0.349 | 2.040 | 0.041 |
| 26946 | TRPC7 | 72.417 | 0.718 | 0.348 | 2.062 | 0.039 |
| 214804 | SYDE2 | 73.661 | 0.723 | 0.342 | 2.114 | 0.034 |
| 216292 | METTL25 | 48.108 | 0.725 | 0.364 | 1.993 | 0.046 |
| 207474 | Kctd12b | 68.980 | 0.725 | 0.330 | 2.197 | 0.028 |
| 22778 | IKZF1 | 70.143 | 0.727 | 0.334 | 2.174 | 0.030 |
| 104099 | ITGA9 | 118.051 | 0.727 | 0.364 | 1.996 | 0.046 |
| 387285 | HCRTR2 | 56.582 | 0.734 | 0.349 | 2.105 | 0.035 |
| 330323 | FAM188B | 74.441 | 0.735 | 0.363 | 2.025 | 0.043 |
| 13607 | EDA | 78.165 | 0.739 | 0.353 | 2.092 | 0.036 |
| 245902 | ccdc15 | 64.668 | 0.740 | 0.322 | 2.296 | 0.022 |
| 15957 | IFIT1 | 58.691 | 0.744 | 0.371 | 2.002 | 0.045 |
| 218973 | WDHD1 | 68.987 | 0.744 | 0.357 | 2.083 | 0.037 |
| 13733 | ADGRE1 | 62.176 | 0.746 | 0.348 | 2.146 | 0.032 |
| 18700 | PIGA | 57.844 | 0.748 | 0.342 | 2.188 | 0.029 |
| 235527 | PLSCR4 | 53.179 | 0.748 | 0.370 | 2.020 | 0.043 |
| 18826 | LCP1 | 99.566 | 0.749 | 0.297 | 2.520 | 0.012 |
| 23876 | FBLN5 | 73.757 | 0.751 | 0.373 | 2.016 | 0.044 |
| 97165 | HMGB2 | 56.607 | 0.754 | 0.366 | 2.060 | 0.039 |
| 73713 | RBM20 | 86.789 | 0.756 | 0.372 | 2.031 | 0.042 |
| 22779 | IKZF2 | 113.516 | 0.758 | 0.359 | 2.112 | 0.035 |
| 237436 | GAS2L3 | 72.298 | 0.759 | 0.369 | 2.056 | 0.040 |
| 20750 | SPP1 | 106.098 | 0.759 | 0.364 | 2.084 | 0.037 |
| 18968 | POLA1 | 123.357 | 0.761 | 0.318 | 2.392 | 0.017 |
| 67547 | slc39a8 | 54.680 | 0.766 | 0.354 | 2.164 | 0.030 |
| 242050 | IGSF10 | 103.948 | 0.773 | 0.356 | 2.174 | 0.030 |
| 21336 | TACR1 | 73.272 | 0.774 | 0.361 | 2.141 | 0.032 |
| 103080 | 10-Sep | 48.417 | 0.775 | 0.378 | 2.046 | 0.041 |
| 215798 | ADGRG6 | 83.309 | 0.777 | 0.394 | 1.971 | 0.049 |
| 70779 | PRDM5 | 63.629 | 0.777 | 0.339 | 2.293 | 0.022 |
| 27401 | SKP2 | 100.887 | 0.782 | 0.328 | 2.388 | 0.017 |
| 98267 | STK17B | 75.168 | 0.783 | 0.312 | 2.510 | 0.012 |
| 13406 | DMP1 | 44.647 | 0.783 | 0.398 | 1.970 | 0.049 |
| 18132 | NOTCH4 | 95.679 | 0.786 | 0.374 | 2.102 | 0.036 |
| 66011 | RANBP17 | 101.049 | 0.786 | 0.334 | 2.356 | 0.018 |
| 14051 | EYA4 | 75.679 | 0.789 | 0.345 | 2.288 | 0.022 |
| 54670 | ATP8B1 | 90.462 | 0.794 | 0.399 | 1.989 | 0.047 |
| 83433 | TREM2 | 53.594 | 0.795 | 0.369 | 2.156 | 0.031 |
| 80285 | PARP9 | 51.829 | 0.797 | 0.384 | 2.076 | 0.038 |
| 19011 | ENDOU | 42.391 | 0.798 | 0.399 | 1.998 | 0.046 |
| 15203 | HEPH | 96.091 | 0.802 | 0.374 | 2.144 | 0.032 |
| 320606 | 3632454L22Rik | 59.660 | 0.804 | 0.406 | 1.981 | 0.048 |
| 224008 | SPIDR | 54.156 | 0.808 | 0.394 | 2.049 | 0.040 |
| 53945 | SLC40A1 | 58.165 | 0.809 | 0.365 | 2.217 | 0.027 |
| 13982 | ESR1 | 56.763 | 0.812 | 0.368 | 2.206 | 0.027 |
| 76223 | AGBL3 | 68.206 | 0.813 | 0.333 | 2.438 | 0.015 |
| 353282 | SFMBT2 | 128.502 | 0.819 | 0.382 | 2.146 | 0.032 |
| 17533 | MRC1 | 81.196 | 0.822 | 0.364 | 2.256 | 0.024 |
| 228913 | Zfp217 | 70.697 | 0.823 | 0.416 | 1.979 | 0.048 |
| 14089 | FAP | 52.925 | 0.824 | 0.407 | 2.024 | 0.043 |
| 20928 | ABCC9 | 121.138 | 0.828 | 0.369 | 2.245 | 0.025 |
| 109904 | MCF2 | 103.982 | 0.829 | 0.299 | 2.775 | 0.006 |
| 14069 | F8 | 118.186 | 0.829 | 0.323 | 2.567 | 0.010 |
| 11629 | AIF1 | 39.798 | 0.829 | 0.423 | 1.961 | 0.050 |
| 30955 | PIK3CG | 92.567 | 0.831 | 0.346 | 2.402 | 0.016 |
| 13482 | DPP4 | 99.800 | 0.834 | 0.332 | 2.513 | 0.012 |
| 19264 | PTPRC | 76.614 | 0.834 | 0.385 | 2.167 | 0.030 |
| 240638 | SLC16A12 | 54.023 | 0.834 | 0.392 | 2.129 | 0.033 |
| 18414 | OSMR | 84.646 | 0.834 | 0.359 | 2.324 | 0.020 |
| 20618 | SNCG | 58.393 | 0.836 | 0.379 | 2.203 | 0.028 |
| 72823 | PARD3B | 110.974 | 0.836 | 0.370 | 2.260 | 0.024 |
| 23794 | ADAMTS5 | 85.840 | 0.837 | 0.380 | 2.203 | 0.028 |
| 75767 | RAB11FIP1 | 82.082 | 0.838 | 0.410 | 2.041 | 0.041 |
| 75746 | MORC4 | 63.396 | 0.839 | 0.387 | 2.165 | 0.030 |
| 21853 | TIMELESS | 77.427 | 0.840 | 0.426 | 1.970 | 0.049 |
| 331374 | DGKK | 104.077 | 0.841 | 0.364 | 2.310 | 0.021 |
| 71909 | HAUS5 | 42.590 | 0.843 | 0.418 | 2.015 | 0.044 |
| 18783 | PLA2G4A | 50.505 | 0.844 | 0.380 | 2.222 | 0.026 |
| 269959 | ADAMTSL3 | 82.871 | 0.847 | 0.403 | 2.100 | 0.036 |
| 240660 | SLC35G1 | 43.008 | 0.847 | 0.420 | 2.016 | 0.044 |
| 109242 | KIF24 | 69.044 | 0.847 | 0.398 | 2.128 | 0.033 |
| 78257 | lrrc9 | 75.444 | 0.848 | 0.378 | 2.243 | 0.025 |
| 66388 | CUTC | 37.878 | 0.852 | 0.423 | 2.015 | 0.044 |
| 16772 | LAMA1 | 139.637 | 0.857 | 0.398 | 2.154 | 0.031 |
| 16985 | LSP1 | 40.632 | 0.857 | 0.415 | 2.068 | 0.039 |
| 12394 | RUNX1 | 78.022 | 0.858 | 0.432 | 1.986 | 0.047 |
| 94176 | DOCK2 | 125.962 | 0.860 | 0.384 | 2.237 | 0.025 |
| 18669 | Abcb1b | 80.375 | 0.861 | 0.408 | 2.107 | 0.035 |
| 547253 | PARP14 | 99.311 | 0.861 | 0.365 | 2.356 | 0.018 |
| 17997 | NEDD1 | 64.665 | 0.861 | 0.390 | 2.206 | 0.027 |
| 17152 | MAK | 60.235 | 0.862 | 0.381 | 2.265 | 0.024 |
| 74044 | TTF2 | 60.162 | 0.868 | 0.439 | 1.980 | 0.048 |
| 68010 | BAMBI | 63.972 | 0.869 | 0.346 | 2.508 | 0.012 |
| 330502 | ZFP82 | 60.019 | 0.870 | 0.394 | 2.210 | 0.027 |
| 319767 | ATP10B | 84.842 | 0.875 | 0.414 | 2.115 | 0.034 |
| 100124453 | Mir654 | 1.204 | 0.876 | 0.445 | 1.970 | 0.049 |
| 72230 | Zfp558 | 58.277 | 0.877 | 0.378 | 2.317 | 0.020 |
| 244049 | MCTP2 | 79.701 | 0.877 | 0.428 | 2.050 | 0.040 |
| 723919 | MIR452 | 1.048 | 0.879 | 0.445 | 1.974 | 0.048 |
| 723948 | MIR302B | 1.048 | 0.879 | 0.445 | 1.974 | 0.048 |
| 100628621 | Mir3961 | 1.048 | 0.879 | 0.445 | 1.974 | 0.048 |
| 18198 | MUSK | 49.370 | 0.879 | 0.436 | 2.019 | 0.043 |
| 229898 | GBP5 | 46.544 | 0.881 | 0.394 | 2.238 | 0.025 |
| 408065 | Zfp456 | 44.474 | 0.881 | 0.445 | 1.978 | 0.048 |
| 12319 | car8 | 155.071 | 0.881 | 0.288 | 3.064 | 0.002 |
| 71724 | AOX3 | 79.308 | 0.883 | 0.404 | 2.186 | 0.029 |
| 26432 | PLOD2 | 62.096 | 0.884 | 0.337 | 2.624 | 0.009 |
| 110082 | DNAH5 | 223.188 | 0.887 | 0.401 | 2.214 | 0.027 |
| 16551 | KIF11 | 58.104 | 0.888 | 0.451 | 1.969 | 0.049 |
| 67454 | IKBIP | 93.236 | 0.888 | 0.293 | 3.030 | 0.002 |
| 209268 | IGSF1 | 91.393 | 0.890 | 0.371 | 2.399 | 0.016 |
| 107815 | SCML2 | 54.595 | 0.890 | 0.452 | 1.971 | 0.049 |
| 23850 | PAPPA2 | 98.300 | 0.892 | 0.417 | 2.140 | 0.032 |
| 74978 | LRRIQ1 | 92.466 | 0.893 | 0.340 | 2.629 | 0.009 |
| 245583 | Tgif2lx1 | 0.963 | 0.893 | 0.447 | 1.996 | 0.046 |
| 67009 | TTC23 | 72.738 | 0.893 | 0.358 | 2.493 | 0.013 |
| 404740 | IGLJ3 | 1.550 | 0.893 | 0.446 | 2.002 | 0.045 |
| 408066 | BC067074 | 88.715 | 0.894 | 0.404 | 2.211 | 0.027 |
| 100124496 | Mir743b | 1.896 | 0.895 | 0.446 | 2.007 | 0.045 |
| 626305 | Scgb1b7 | 1.491 | 0.896 | 0.447 | 2.005 | 0.045 |
| 101401 | ADAMTS9 | 83.862 | 0.897 | 0.419 | 2.141 | 0.032 |
| 385354 | FRMD7 | 61.505 | 0.899 | 0.414 | 2.168 | 0.030 |
| 20128 | Trim30a | 48.804 | 0.901 | 0.408 | 2.206 | 0.027 |
| 319739 | B230303O12Rik | 52.303 | 0.902 | 0.454 | 1.986 | 0.047 |
| 100040885 | Gm16427 | 1.238 | 0.902 | 0.448 | 2.014 | 0.044 |
| 16780 | LAMB3 | 53.809 | 0.904 | 0.452 | 1.999 | 0.046 |
| 70348 | Ube2cbp | 38.126 | 0.905 | 0.425 | 2.128 | 0.033 |
| 19650 | RBL1 | 72.739 | 0.905 | 0.419 | 2.162 | 0.031 |
| 71924 | TUBE1 | 42.016 | 0.907 | 0.432 | 2.097 | 0.036 |
| 22310 | Vmn2r42 | 1.394 | 0.907 | 0.449 | 2.022 | 0.043 |
| 381072 | Abca17 | 58.785 | 0.908 | 0.455 | 1.996 | 0.046 |
| 272636 | ESYT3 | 72.374 | 0.909 | 0.437 | 2.081 | 0.037 |
| 17427 | MNS1 | 35.908 | 0.909 | 0.439 | 2.070 | 0.038 |
| 330096 | SHISA3 | 40.655 | 0.910 | 0.456 | 1.998 | 0.046 |
| 227058 | Dnah7b | 158.669 | 0.911 | 0.407 | 2.240 | 0.025 |
| 60440 | iigp1 | 37.627 | 0.911 | 0.449 | 2.031 | 0.042 |
| 239839 | CCDC14 | 51.644 | 0.912 | 0.413 | 2.207 | 0.027 |
| 330149 | HFM1 | 81.004 | 0.912 | 0.408 | 2.233 | 0.026 |
| 70005 | Znf41-ps | 43.930 | 0.912 | 0.426 | 2.139 | 0.032 |
| 100039763 | Gm2411 | 2.086 | 0.913 | 0.448 | 2.038 | 0.042 |
| 97086 | SLC9B2 | 43.316 | 0.914 | 0.454 | 2.014 | 0.044 |
| 619303 | 4930567H17Rik | 1.740 | 0.915 | 0.449 | 2.039 | 0.041 |
| 100862015 | Gm11236 | 1.740 | 0.915 | 0.449 | 2.039 | 0.041 |
| 64929 | scel | 62.476 | 0.916 | 0.400 | 2.291 | 0.022 |
| 229214 | QRFPR | 35.495 | 0.917 | 0.429 | 2.135 | 0.033 |
| 229357 | GPR149 | 49.882 | 0.917 | 0.380 | 2.415 | 0.016 |
| 100861685 | Gm21135 | 1.968 | 0.921 | 0.449 | 2.049 | 0.040 |
| 109245 | LRRC39 | 60.286 | 0.921 | 0.451 | 2.043 | 0.041 |
| 329872 | FREM1 | 122.522 | 0.921 | 0.392 | 2.347 | 0.019 |
| 17916 | MYO1F | 62.420 | 0.923 | 0.403 | 2.287 | 0.022 |
| 209387 | Trim30d | 49.193 | 0.926 | 0.460 | 2.014 | 0.044 |
| 751550 | MIR146B | 1.179 | 0.928 | 0.452 | 2.052 | 0.040 |
| 20765 | Sprr2k | 7.131 | 0.929 | 0.470 | 1.975 | 0.048 |
| 100504506 | Gm12891 | 1.774 | 0.930 | 0.451 | 2.063 | 0.039 |
| 258060 | Olfr1181 | 6.813 | 0.930 | 0.472 | 1.971 | 0.049 |
| 100038948 | Mup9 | 3.315 | 0.932 | 0.449 | 2.074 | 0.038 |
| 105120 | AI197445 | 60.645 | 0.933 | 0.438 | 2.129 | 0.033 |
| 12817 | COL13A1 | 62.574 | 0.933 | 0.440 | 2.118 | 0.034 |
| 74580 | PYROXD2 | 46.127 | 0.934 | 0.458 | 2.038 | 0.042 |
| 780931 | Ighv1-53 | 3.066 | 0.934 | 0.450 | 2.076 | 0.038 |
| 319727 | A330035P11Rik | 37.462 | 0.934 | 0.474 | 1.971 | 0.049 |
| 626055 | Gm15645 | 1.681 | 0.936 | 0.452 | 2.070 | 0.038 |
| 629785 | Gm7003 | 1.681 | 0.936 | 0.452 | 2.070 | 0.038 |
| 100040771 | Gm2959 | 1.681 | 0.936 | 0.452 | 2.070 | 0.038 |
| 436230 | BC065397 | 46.977 | 0.937 | 0.415 | 2.260 | 0.024 |
| 791309 | Gm9919 | 40.479 | 0.938 | 0.458 | 2.047 | 0.041 |
| 11979 | ATP7B | 76.195 | 0.944 | 0.433 | 2.180 | 0.029 |
| 72208 | 1700016L21Rik | 4.081 | 0.945 | 0.470 | 2.012 | 0.044 |
| 432628 | MFSD2B | 42.759 | 0.946 | 0.475 | 1.994 | 0.046 |
| 626596 | RGS22 | 56.542 | 0.949 | 0.437 | 2.174 | 0.030 |
| 246102 | RTTN | 132.757 | 0.949 | 0.345 | 2.750 | 0.006 |
| 66864 | CLEC14A | 65.500 | 0.950 | 0.350 | 2.715 | 0.007 |
| 382695 | Ighv1-56 | 3.349 | 0.951 | 0.452 | 2.104 | 0.035 |
| 208431 | SHROOM4 | 80.167 | 0.954 | 0.364 | 2.617 | 0.009 |
| 74249 | LRRC2 | 46.054 | 0.956 | 0.444 | 2.154 | 0.031 |
| 68760 | SYNPO2L | 35.079 | 0.957 | 0.472 | 2.027 | 0.043 |
| 387176 | MIR181A-2 | 2.158 | 0.958 | 0.454 | 2.110 | 0.035 |
| 75424 | Zfp820 | 43.708 | 0.960 | 0.466 | 2.059 | 0.039 |
| 170939 | Krtap19-9b | 4.300 | 0.960 | 0.471 | 2.039 | 0.041 |
| 117591 | SLC2A9 | 51.092 | 0.963 | 0.476 | 2.023 | 0.043 |
| 12649 | CHEK1 | 49.719 | 0.963 | 0.437 | 2.203 | 0.028 |
| 238427 | Ighv6-6 | 1.715 | 0.964 | 0.456 | 2.117 | 0.034 |
| 545370 | HMCN1 | 279.181 | 0.964 | 0.438 | 2.203 | 0.028 |
| 56788 | SCUBE2 | 57.939 | 0.965 | 0.444 | 2.172 | 0.030 |
| 320662 | CASC1 | 34.264 | 0.965 | 0.484 | 1.995 | 0.046 |
| 791299 | Gm10069 | 45.454 | 0.966 | 0.411 | 2.349 | 0.019 |
| 50706 | POSTN | 62.503 | 0.966 | 0.428 | 2.257 | 0.024 |
| 16952 | ANXA1 | 30.164 | 0.967 | 0.467 | 2.071 | 0.038 |
| 381693 | Wdr95 | 39.554 | 0.968 | 0.477 | 2.030 | 0.042 |
| 74726 | 4930523O13Rik | 15.265 | 0.969 | 0.494 | 1.962 | 0.050 |
| 319625 | GALM | 33.298 | 0.970 | 0.491 | 1.977 | 0.048 |
| 17202 | MC4R | 32.437 | 0.970 | 0.471 | 2.062 | 0.039 |
| 108022 | Igkv6-15 | 3.870 | 0.971 | 0.474 | 2.048 | 0.041 |
| 240892 | DUSP27 | 43.542 | 0.971 | 0.492 | 1.973 | 0.048 |
| 381067 | Zfp229 | 75.361 | 0.972 | 0.312 | 3.120 | 0.002 |
| 240322 | ADAMTS19 | 55.126 | 0.974 | 0.461 | 2.112 | 0.035 |
| 100504448 | Gm20234 | 2.748 | 0.975 | 0.470 | 2.075 | 0.038 |
| 269954 | TTLL13 | 43.846 | 0.975 | 0.473 | 2.059 | 0.039 |
| 381924 | ITGAD | 42.908 | 0.975 | 0.492 | 1.981 | 0.048 |
| 19260 | PTPN22 | 42.110 | 0.976 | 0.491 | 1.990 | 0.047 |
| 105837 | MTBP | 67.686 | 0.978 | 0.425 | 2.300 | 0.021 |
| 14425 | GALNT3 | 45.244 | 0.978 | 0.439 | 2.229 | 0.026 |
| 319472 | 9330158H04Rik | 28.953 | 0.978 | 0.496 | 1.973 | 0.048 |
| 27214 | DBF4 | 37.318 | 0.980 | 0.459 | 2.134 | 0.033 |
| 259000 | Olfr195 | 4.490 | 0.981 | 0.473 | 2.076 | 0.038 |
| 215387 | NCAPH | 45.043 | 0.982 | 0.452 | 2.172 | 0.030 |
| 227929 | CYTIP | 42.977 | 0.982 | 0.497 | 1.976 | 0.048 |
| 259019 | Olfr1045 | 7.227 | 0.983 | 0.492 | 2.000 | 0.046 |
| 257935 | Olfr1287 | 6.451 | 0.983 | 0.479 | 2.052 | 0.040 |
| 104271 | TEX15 | 94.202 | 0.984 | 0.395 | 2.493 | 0.013 |
| 93671 | CD163 | 71.045 | 0.985 | 0.418 | 2.356 | 0.018 |
| 320163 | 4930525G20Rik | 34.516 | 0.985 | 0.457 | 2.154 | 0.031 |
| 240697 | MCMDC2 | 53.646 | 0.986 | 0.399 | 2.472 | 0.013 |
| 320159 | FAM179A | 56.777 | 0.988 | 0.458 | 2.158 | 0.031 |
| 780802 | ighv4-1 | 1.846 | 0.988 | 0.458 | 2.156 | 0.031 |
| 22138 | TTN | 1128.360 | 0.989 | 0.503 | 1.967 | 0.049 |
| 77945 | RPGRIP1 | 53.215 | 0.989 | 0.470 | 2.106 | 0.035 |
| 18285 | ODF1 | 10.918 | 0.991 | 0.496 | 1.999 | 0.046 |
| 67121 | MASTL | 55.709 | 0.992 | 0.436 | 2.275 | 0.023 |
| 18505 | PAX3 | 35.790 | 0.992 | 0.503 | 1.972 | 0.049 |
| 319764 | A730046J19Rik | 42.931 | 0.992 | 0.489 | 2.028 | 0.043 |
| 218518 | MARVELD2 | 38.475 | 0.993 | 0.501 | 1.981 | 0.048 |
| 14537 | GCNT1 | 68.582 | 0.993 | 0.337 | 2.948 | 0.003 |
| 76668 | MDH1B | 28.623 | 0.994 | 0.504 | 1.974 | 0.048 |
| 71872 | AOX4 | 70.896 | 0.995 | 0.422 | 2.357 | 0.018 |
| 327992 | hsf5 | 36.853 | 0.995 | 0.488 | 2.037 | 0.042 |
| 407809 | BC055402 | 37.335 | 0.996 | 0.505 | 1.975 | 0.048 |
| 56794 | HACL1 | 50.943 | 0.998 | 0.375 | 2.664 | 0.008 |
| 258782 | Olfr914 | 6.274 | 0.999 | 0.482 | 2.074 | 0.038 |
| 330721 | NEK5 | 37.735 | 0.999 | 0.496 | 2.014 | 0.044 |
| 545848 | Igkv4-80 | 2.797 | 1.000 | 0.458 | 2.182 | 0.029 |
| 76670 | CFAP70 | 63.321 | 1.001 | 0.444 | 2.255 | 0.024 |
| 442799 | 9330154J02Rik | 26.289 | 1.002 | 0.507 | 1.977 | 0.048 |
| 56552 | Vmn2r26 | 19.325 | 1.003 | 0.509 | 1.970 | 0.049 |
| 73278 | 1700031P21Rik | 3.582 | 1.003 | 0.458 | 2.190 | 0.029 |
| 22412 | WNT9B | 41.671 | 1.003 | 0.472 | 2.126 | 0.034 |
| 66977 | NUF2 | 32.920 | 1.003 | 0.464 | 2.162 | 0.031 |
| 257889 | Olfr132 | 8.345 | 1.004 | 0.493 | 2.037 | 0.042 |
| 56419 | DIAPH3 | 53.277 | 1.004 | 0.467 | 2.151 | 0.031 |
| 16364 | IRF4 | 44.998 | 1.009 | 0.478 | 2.112 | 0.035 |
| 20147 | RS1 | 59.923 | 1.009 | 0.431 | 2.341 | 0.019 |
| 108670 | EPSTI1 | 29.410 | 1.011 | 0.495 | 2.044 | 0.041 |
| 218581 | depdc1b | 29.536 | 1.011 | 0.511 | 1.977 | 0.048 |
| 70454 | CENPL | 45.682 | 1.011 | 0.452 | 2.236 | 0.025 |
| 66722 | SPAG16 | 37.334 | 1.012 | 0.476 | 2.126 | 0.034 |
| 270672 | MAP3K15 | 56.536 | 1.015 | 0.445 | 2.279 | 0.023 |
| 319812 | 9430018G01Rik | 34.761 | 1.015 | 0.508 | 1.998 | 0.046 |
| 233424 | TMC3 | 55.146 | 1.015 | 0.423 | 2.400 | 0.016 |
| 381112 | ARHGEF33 | 43.911 | 1.018 | 0.465 | 2.188 | 0.029 |
| 77782 | POLQ | 107.460 | 1.018 | 0.382 | 2.663 | 0.008 |
| 56094 | Cts8 | 20.943 | 1.019 | 0.519 | 1.964 | 0.050 |
| 12571 | CDK6 | 33.198 | 1.019 | 0.482 | 2.114 | 0.035 |
| 17215 | MCM3 | 54.220 | 1.020 | 0.427 | 2.386 | 0.017 |
| 331046 | TGM4 | 42.197 | 1.021 | 0.483 | 2.111 | 0.035 |
| 329093 | cpa6 | 25.200 | 1.021 | 0.516 | 1.978 | 0.048 |
| 71398 | 5430427O19Rik | 37.015 | 1.021 | 0.513 | 1.992 | 0.046 |
| 434036 | Igkv13-85 | 3.421 | 1.022 | 0.460 | 2.219 | 0.026 |
| 67141 | FBXO5 | 23.836 | 1.022 | 0.513 | 1.994 | 0.046 |
| 100504653 | 4930555A03Rik | 19.684 | 1.023 | 0.520 | 1.969 | 0.049 |
| 16184 | IL2RA | 38.631 | 1.024 | 0.500 | 2.047 | 0.041 |
| 75033 | MEI4 | 36.480 | 1.025 | 0.466 | 2.199 | 0.028 |
| 140498 | RXFP2 | 44.743 | 1.025 | 0.489 | 2.096 | 0.036 |
| 11609 | AGTR2 | 31.747 | 1.025 | 0.489 | 2.096 | 0.036 |
| 237178 | PPEF1 | 39.004 | 1.026 | 0.505 | 2.032 | 0.042 |
| 22355 | VIPR2 | 58.871 | 1.026 | 0.425 | 2.414 | 0.016 |
| 66611 | RIBC1 | 22.243 | 1.026 | 0.517 | 1.984 | 0.047 |
| 214111 | SLC24A1 | 59.004 | 1.028 | 0.446 | 2.303 | 0.021 |
| 625286 | TMEM236 | 32.040 | 1.028 | 0.519 | 1.981 | 0.048 |
| 214763 | MB21D1 | 45.854 | 1.028 | 0.459 | 2.239 | 0.025 |
| 73246 | RASSF6 | 28.267 | 1.030 | 0.501 | 2.057 | 0.040 |
| 271697 | CDK15 | 22.363 | 1.030 | 0.516 | 1.997 | 0.046 |
| 432552 | FAM71B | 18.328 | 1.032 | 0.519 | 1.988 | 0.047 |
| 12267 | C3AR1 | 54.318 | 1.033 | 0.363 | 2.848 | 0.004 |
| 242838 | LRRD1 | 27.466 | 1.033 | 0.518 | 1.995 | 0.046 |
| 66442 | SPC25 | 25.283 | 1.034 | 0.516 | 2.003 | 0.045 |
| 170722 | Nxf7 | 37.166 | 1.035 | 0.498 | 2.079 | 0.038 |
| 17857 | MX1 | 32.471 | 1.035 | 0.512 | 2.022 | 0.043 |
| 66745 | Trpd52l3 | 22.270 | 1.035 | 0.520 | 1.989 | 0.047 |
| 277328 | TRPA1 | 47.299 | 1.036 | 0.489 | 2.120 | 0.034 |
| 666803 | Gm8298 | 14.606 | 1.038 | 0.509 | 2.039 | 0.041 |
| 436240 | FOXR2 | 27.761 | 1.038 | 0.499 | 2.080 | 0.038 |
| 258533 | Olfr1364 | 9.502 | 1.039 | 0.492 | 2.110 | 0.035 |
| 213234 | zbbx | 41.059 | 1.039 | 0.440 | 2.362 | 0.018 |
| 545126 | CN725425 | 21.115 | 1.040 | 0.521 | 1.997 | 0.046 |
| 17996 | NEB | 311.656 | 1.040 | 0.462 | 2.253 | 0.024 |
| 100504191 | Gm16576 | 53.533 | 1.040 | 0.469 | 2.219 | 0.026 |
| 622921 | Gm6369 | 4.394 | 1.040 | 0.479 | 2.171 | 0.030 |
| 22038 | PLSCR1 | 32.165 | 1.041 | 0.474 | 2.198 | 0.028 |
| 69693 | POF1B | 46.463 | 1.043 | 0.482 | 2.165 | 0.030 |
| 382044 | Ces1b | 22.589 | 1.044 | 0.519 | 2.012 | 0.044 |
| 76184 | ABCA6 | 90.291 | 1.044 | 0.415 | 2.514 | 0.012 |
| 347708 | dppA1 | 19.157 | 1.046 | 0.520 | 2.010 | 0.044 |
| 68792 | SRPX2 | 39.152 | 1.049 | 0.467 | 2.246 | 0.025 |
| 380660 | ACSS3 | 35.837 | 1.049 | 0.478 | 2.193 | 0.028 |
| 243085 | Ugt2b35 | 24.974 | 1.049 | 0.520 | 2.016 | 0.044 |
| 232078 | THNSL2 | 38.124 | 1.052 | 0.460 | 2.286 | 0.022 |
| 16192 | IL5RA | 41.968 | 1.052 | 0.503 | 2.093 | 0.036 |
| 20526 | SLC2A2 | 31.582 | 1.053 | 0.520 | 2.024 | 0.043 |
| 271508 | 4933408B17Rik | 23.132 | 1.053 | 0.518 | 2.032 | 0.042 |
| 100043462 | Nutf2-ps1 | 22.400 | 1.054 | 0.507 | 2.078 | 0.038 |
| 75084 | 4930511M06Rik | 32.216 | 1.054 | 0.509 | 2.070 | 0.038 |
| 72361 | Ces2g | 31.380 | 1.055 | 0.506 | 2.085 | 0.037 |
| 14380 | G6pd2 | 12.996 | 1.059 | 0.509 | 2.080 | 0.037 |
| 381476 | STPG2 | 35.768 | 1.059 | 0.516 | 2.055 | 0.040 |
| 258875 | Olfr895 | 7.529 | 1.060 | 0.492 | 2.152 | 0.031 |
| 100504728 | Gm13985 | 19.928 | 1.060 | 0.518 | 2.047 | 0.041 |
| 170639 | Olfr78 | 34.992 | 1.060 | 0.498 | 2.128 | 0.033 |
| 68723 | HRNR | 32.471 | 1.060 | 0.519 | 2.043 | 0.041 |
| 227998 | 4933409G03Rik | 22.401 | 1.062 | 0.517 | 2.052 | 0.040 |
| 72804 | 9130017K11Rik | 30.347 | 1.062 | 0.488 | 2.176 | 0.030 |
| 320671 | D130079A08Rik | 35.950 | 1.062 | 0.507 | 2.094 | 0.036 |
| 56410 | CBLN3 | 62.717 | 1.062 | 0.441 | 2.412 | 0.016 |
| 382053 | Ces3a | 23.342 | 1.063 | 0.519 | 2.047 | 0.041 |
| 258425 | Olfr994 | 3.620 | 1.064 | 0.465 | 2.290 | 0.022 |
| 320454 | Tmprss11g | 29.597 | 1.065 | 0.521 | 2.046 | 0.041 |
| 268729 | FRMPD2 | 52.954 | 1.065 | 0.466 | 2.286 | 0.022 |
| 207920 | ESRP1 | 35.557 | 1.065 | 0.513 | 2.077 | 0.038 |
| 11522 | ADH1 | 17.176 | 1.066 | 0.520 | 2.048 | 0.041 |
| 55932 | GBP3 | 58.391 | 1.066 | 0.334 | 3.188 | 0.001 |
| 171188 | Vmn1r32 | 20.329 | 1.067 | 0.520 | 2.050 | 0.040 |
| 11923 | NEUROD4 | 23.340 | 1.067 | 0.514 | 2.074 | 0.038 |
| 232748 | TCAF2 | 42.052 | 1.068 | 0.493 | 2.165 | 0.030 |
| 71887 | PPM1J | 24.948 | 1.068 | 0.519 | 2.060 | 0.039 |
| 56872 | PATE4 | 13.693 | 1.069 | 0.516 | 2.073 | 0.038 |
| 627537 | Vmn2r100 | 19.869 | 1.069 | 0.516 | 2.071 | 0.038 |
| 71862 | gpr160 | 25.316 | 1.071 | 0.502 | 2.132 | 0.033 |
| 11752 | ANXA8 | 27.311 | 1.072 | 0.511 | 2.098 | 0.036 |
| 448987 | FBXL7 | 46.084 | 1.072 | 0.436 | 2.457 | 0.014 |
| 16948 | LOX | 30.331 | 1.073 | 0.506 | 2.122 | 0.034 |
| 636104 | Gm7173 | 44.093 | 1.074 | 0.467 | 2.298 | 0.022 |
| 13661 | EHF | 40.904 | 1.074 | 0.505 | 2.127 | 0.033 |
| 353025 | CAPS2 | 33.030 | 1.075 | 0.514 | 2.091 | 0.037 |
| 12236 | BUB1B | 57.575 | 1.078 | 0.444 | 2.430 | 0.015 |
| 319430 | C5AR2 | 24.813 | 1.078 | 0.513 | 2.103 | 0.035 |
| 12364 | CASP12 | 33.579 | 1.079 | 0.478 | 2.255 | 0.024 |
| 12814 | COL11A1 | 134.483 | 1.079 | 0.398 | 2.715 | 0.007 |
| 12902 | CR2 | 64.462 | 1.081 | 0.430 | 2.512 | 0.012 |
| 22177 | TYROBP | 69.592 | 1.081 | 0.413 | 2.617 | 0.009 |
| 74441 | Slco6c1 | 28.381 | 1.083 | 0.520 | 2.082 | 0.037 |
| 17076 | LY75 | 62.180 | 1.084 | 0.448 | 2.417 | 0.016 |
| 399570 | Kank4os | 28.176 | 1.085 | 0.519 | 2.089 | 0.037 |
| 108812 | ALS2CR12 | 28.742 | 1.085 | 0.520 | 2.086 | 0.037 |
| 15950 | Ifi203 | 37.674 | 1.085 | 0.482 | 2.251 | 0.024 |
| 414801 | ITPRIP | 34.740 | 1.087 | 0.487 | 2.234 | 0.026 |
| 627984 | Nlrp1c-ps | 38.716 | 1.087 | 0.491 | 2.216 | 0.027 |
| 328839 | Gm5094 | 22.224 | 1.088 | 0.520 | 2.093 | 0.036 |
| 241612 | SLC5A12 | 56.837 | 1.089 | 0.463 | 2.354 | 0.019 |
| 17929 | MYOM1 | 76.244 | 1.093 | 0.419 | 2.610 | 0.009 |
| 70897 | FAM71D | 24.605 | 1.093 | 0.519 | 2.107 | 0.035 |
| 100503044 | A730020M07Rik | 29.154 | 1.095 | 0.498 | 2.200 | 0.028 |
| 18416 | OTC | 26.035 | 1.095 | 0.520 | 2.105 | 0.035 |
| 626870 | Gm11992 | 22.413 | 1.096 | 0.509 | 2.153 | 0.031 |
| 110855 | PDE6C | 34.077 | 1.097 | 0.506 | 2.169 | 0.030 |
| 212377 | MMS22L | 66.482 | 1.097 | 0.443 | 2.477 | 0.013 |
| 226049 | DMRT2 | 27.330 | 1.100 | 0.509 | 2.161 | 0.031 |
| 12180 | SMYD1 | 48.051 | 1.100 | 0.391 | 2.814 | 0.005 |
| 13206 | DDX4 | 46.495 | 1.102 | 0.461 | 2.391 | 0.017 |
| 69129 | PEX11G | 18.467 | 1.102 | 0.518 | 2.129 | 0.033 |
| 279572 | tlr13 | 44.463 | 1.102 | 0.400 | 2.755 | 0.006 |
| 75822 | 4930524C18Rik | 18.597 | 1.105 | 0.519 | 2.128 | 0.033 |
| 73809 | SATL1 | 26.786 | 1.108 | 0.520 | 2.131 | 0.033 |
| 73093 | 3110006O06Rik | 40.815 | 1.110 | 0.472 | 2.353 | 0.019 |
| 23828 | BVES | 24.814 | 1.111 | 0.513 | 2.165 | 0.030 |
| 692161 | Igkv19-93 | 4.332 | 1.111 | 0.478 | 2.324 | 0.020 |
| 100504235 | Gm20125 | 31.657 | 1.112 | 0.519 | 2.143 | 0.032 |
| 12523 | CD84 | 84.762 | 1.114 | 0.368 | 3.026 | 0.002 |
| 16545 | KERA | 15.809 | 1.115 | 0.517 | 2.156 | 0.031 |
| 232406 | BC035044 | 34.758 | 1.117 | 0.490 | 2.282 | 0.023 |
| 258550 | Olfr869 | 8.254 | 1.118 | 0.488 | 2.293 | 0.022 |
| 74145 | F13A1 | 54.077 | 1.119 | 0.405 | 2.766 | 0.006 |
| 76131 | depdc1a | 29.998 | 1.120 | 0.520 | 2.155 | 0.031 |
| 74405 | EFHC2 | 49.046 | 1.121 | 0.435 | 2.575 | 0.010 |
| 258835 | Olfr1130 | 7.205 | 1.122 | 0.507 | 2.212 | 0.027 |
| 270711 | FAM26D | 30.760 | 1.122 | 0.521 | 2.155 | 0.031 |
| 245533 | AWAT1 | 21.989 | 1.126 | 0.520 | 2.166 | 0.030 |
| 241197 | SERPINB10 | 27.143 | 1.126 | 0.521 | 2.163 | 0.031 |
| 105387 | Akr1c14 | 36.237 | 1.128 | 0.479 | 2.353 | 0.019 |
| 15959 | IFIT3 | 60.989 | 1.130 | 0.348 | 3.247 | 0.001 |
| 209590 | IL23R | 25.376 | 1.131 | 0.519 | 2.179 | 0.029 |
| 213248 | WDR49 | 40.611 | 1.137 | 0.475 | 2.395 | 0.017 |
| 78321 | ANKRD23 | 34.899 | 1.137 | 0.477 | 2.385 | 0.017 |
| 236451 | Phf11b | 19.990 | 1.137 | 0.518 | 2.195 | 0.028 |
| 100038713 | Gm10432 | 51.849 | 1.137 | 0.449 | 2.532 | 0.011 |
| 669149 | Vmn2r88 | 20.495 | 1.140 | 0.516 | 2.211 | 0.027 |
| 217578 | BAZ1A | 90.997 | 1.140 | 0.386 | 2.950 | 0.003 |
| 242523 | DMRTA1 | 34.864 | 1.144 | 0.514 | 2.225 | 0.026 |
| 12169 | BMX | 44.962 | 1.144 | 0.463 | 2.474 | 0.013 |
| 74548 | Gsdmc4 | 18.671 | 1.145 | 0.520 | 2.200 | 0.028 |
| 52614 | Adgre4 | 41.488 | 1.146 | 0.505 | 2.269 | 0.023 |
| 211924 | Dsg1c | 30.883 | 1.147 | 0.519 | 2.210 | 0.027 |
| 320022 | TERB1 | 37.742 | 1.148 | 0.462 | 2.483 | 0.013 |
| 225825 | CD226 | 24.492 | 1.149 | 0.520 | 2.209 | 0.027 |
| 75973 | Ccdc162 | 48.851 | 1.151 | 0.483 | 2.381 | 0.017 |
| 14302 | FRK | 40.032 | 1.152 | 0.484 | 2.381 | 0.017 |
| 217653 | MIS18BP1 | 46.963 | 1.156 | 0.466 | 2.481 | 0.013 |
| 12491 | CD36 | 48.368 | 1.158 | 0.460 | 2.520 | 0.012 |
| 70008 | ACE2 | 47.568 | 1.160 | 0.478 | 2.426 | 0.015 |
| 623474 | RAD54B | 45.388 | 1.162 | 0.460 | 2.524 | 0.012 |
| 100042814 | Apol7d | 18.388 | 1.163 | 0.519 | 2.239 | 0.025 |
| 620913 | Gm12185 | 42.926 | 1.164 | 0.494 | 2.357 | 0.018 |
| 83996 | Mmp1b | 25.723 | 1.164 | 0.520 | 2.240 | 0.025 |
| 16197 | IL7R | 36.709 | 1.165 | 0.502 | 2.323 | 0.020 |
| 244237 | Tnfrsf26 | 34.015 | 1.167 | 0.514 | 2.271 | 0.023 |
| 224480 | NOX3 | 29.380 | 1.167 | 0.520 | 2.243 | 0.025 |
| 545477 | Bpifa6 | 19.928 | 1.174 | 0.520 | 2.255 | 0.024 |
| 14013 | MECOM | 70.254 | 1.175 | 0.418 | 2.813 | 0.005 |
| 20849 | STAT4 | 44.468 | 1.177 | 0.449 | 2.621 | 0.009 |
| 258527 | Olfr1368 | 8.967 | 1.178 | 0.483 | 2.440 | 0.015 |
| 81600 | CHIA1 | 31.491 | 1.180 | 0.500 | 2.359 | 0.018 |
| 93837 | DACH2 | 57.508 | 1.183 | 0.458 | 2.583 | 0.010 |
| 404194 | GFRAL | 26.052 | 1.185 | 0.520 | 2.279 | 0.023 |
| 100727 | Ugt2b34 | 25.351 | 1.189 | 0.520 | 2.288 | 0.022 |
| 434197 | FAM169B | 27.058 | 1.189 | 0.517 | 2.299 | 0.022 |
| 13409 | TMC1 | 45.758 | 1.192 | 0.491 | 2.429 | 0.015 |
| 30800 | MMP20 | 34.069 | 1.200 | 0.512 | 2.344 | 0.019 |
| 100041618 | Gm3434 | 6.556 | 1.204 | 0.487 | 2.471 | 0.013 |
| 19113 | Prl7a1 | 12.498 | 1.209 | 0.495 | 2.442 | 0.015 |
| 19051 | PPP1R17 | 24.272 | 1.212 | 0.504 | 2.404 | 0.016 |
| 26888 | Clec4a2 | 31.300 | 1.212 | 0.510 | 2.378 | 0.017 |
| 216343 | TPH2 | 41.367 | 1.212 | 0.432 | 2.808 | 0.005 |
| 244071 | AGBL1 | 48.007 | 1.215 | 0.482 | 2.524 | 0.012 |
| 217593 | SLC25A21 | 36.059 | 1.225 | 0.499 | 2.456 | 0.014 |
| 16769 | DSG4 | 34.819 | 1.226 | 0.519 | 2.364 | 0.018 |
| 12223 | BTC | 24.258 | 1.228 | 0.519 | 2.364 | 0.018 |
| 258513 | Olfr536 | 38.643 | 1.229 | 0.511 | 2.406 | 0.016 |
| 18545 | PCP2 | 21.787 | 1.229 | 0.512 | 2.401 | 0.016 |
| 78250 | IQCH | 52.950 | 1.232 | 0.460 | 2.679 | 0.007 |
| 209334 | GEN1 | 43.696 | 1.247 | 0.485 | 2.569 | 0.010 |
| 19256 | PTPN20 | 38.470 | 1.259 | 0.495 | 2.544 | 0.011 |
| 258741 | Olfr935 | 10.376 | 1.259 | 0.488 | 2.578 | 0.010 |
| 239759 | LIPH | 39.250 | 1.268 | 0.485 | 2.617 | 0.009 |
| 100503240 | TRPC5OS | 37.584 | 1.270 | 0.511 | 2.483 | 0.013 |
| 14399 | GABRA6 | 32.321 | 1.272 | 0.501 | 2.540 | 0.011 |
| 18302 | OIT3 | 35.864 | 1.274 | 0.514 | 2.479 | 0.013 |
| 22061 | Trp63 | 62.267 | 1.294 | 0.429 | 3.015 | 0.003 |
| 258390 | Olfr1276 | 8.306 | 1.297 | 0.500 | 2.592 | 0.010 |
| 22018 | TPO | 43.716 | 1.299 | 0.491 | 2.643 | 0.008 |
| 236312 | Ifi209 | 28.876 | 1.305 | 0.515 | 2.532 | 0.011 |
| 12229 | BTK | 38.637 | 1.321 | 0.501 | 2.639 | 0.008 |
| 240327 | Gm4951 | 25.800 | 1.326 | 0.511 | 2.593 | 0.010 |
| 11571 | CRISP1 | 20.221 | 1.358 | 0.519 | 2.615 | 0.009 |
| 13058 | CYBB | 51.611 | 1.377 | 0.457 | 3.016 | 0.003 |
| 78910 | ASB15 | 36.151 | 1.395 | 0.514 | 2.713 | 0.007 |
| 102920 | CENPI | 44.745 | 1.436 | 0.485 | 2.958 | 0.003 |
| 56644 | CLEC7A | 26.035 | 1.448 | 0.512 | 2.831 | 0.005 |
| 243084 | TMPRSS11E | 32.964 | 1.509 | 0.518 | 2.914 | 0.004 |
|  |  |  |  |  |  |  |
